# Global, Regional, and National Burden of chronic kidney disease in older adults from 1990 to 2021: Results from the Global Burden of Disease Study 2021

**DOI:** 10.1371/journal.pone.0354811

**Published:** 2026-07-31

**Authors:** Weixuan Wang, Junhui Wu, Shaomei Shang

**Affiliations:** School of Nursing, Peking University, Beijing, China; Ministry of Health, Sri Lanka, SRI LANKA

## Abstract

**Background:**

Drawing on data from the Global Burden of Disease (GBD) 2021 study, this research provides the first systematic assessment of the burden and trends of chronic kidney disease (CKD) among the global population aged 75 years and over between 1990 and 2021. Unlike previous studies covering the entire age spectrum, this study focuses on the age group with the highest concentration of disease burden and the greatest health vulnerability, with the aim of revealing the unique epidemiological patterns specific to this population.

**Methods:**

The study methodology involved extracting data on the prevalence, incidence, mortality and disability-adjusted life years (DALYs) for chronic kidney disease (CKD) among people aged 75 years and over in 204 countries and territories from the GBD 2021 database. All reported rates are age-standardized. We calculated estimated annual percentage changes (EAPCs) to analyze trends and examined the association between the disease burden and the Sociodemographic Index (SDI).

**Results:**

The study found that in 2021, approximately 127 million people aged 75 years and over worldwide were living with CKD. Between 1990 and 2021, the age-standardized prevalence declined slightly (EAPC = −0.06%; 95% UI: −0.09% – −0.03%), whilst incidence, mortality and DALY rates all rose significantly (EAPCs of 0.50%; 95% UI: 0.47%–0.53%, 1.55%; 95% UI: 1.48%–1.61% and 1.07%; 95% UI: 1.02%–1.12%, respectively), demonstrating the key phenomenon of ‘prevalence–mortality trend divergence’. The relationship between the burden of disease and socio-economic development is complex: regions with low SDI bear the heaviest burden of mortality, whilst regions with high SDI have experienced the fastest growth in mortality rates. The study also revealed significant heterogeneity in the burden across age, gender and geographical regions.

**Conclusions:**

The burden of chronic kidney disease (CKD) among the global older adult population is rapidly shifting from a pattern of ‘high prevalence’ to one characterised by ‘high incidence, high mortality and high disability’, with the underlying drivers exhibiting fundamental differences across regions at various stages of development. This necessitates that public health strategies move beyond a ‘one-size-fits-all’ approach and instead develop highly age-specific and contextually tailored interventions. This study provides a detailed map for understanding the epidemiology of CKD in the context of extreme population ageing, and offers crucial evidence for the allocation of resources and the formulation of policies.

## Introduction

As the ageing population accelerates and the disease spectrum shifts, older adults are at greater risk of developing chronic kidney disease (CKD), posing a significant health risk and economic burden worldwide [1–6]. CKD is one of the most common chronic diseases worldwide, affecting approximately more than 10% of the global population, and it is projected to become the fifth most important factor affecting life expectancy by 2040 [7,8]. It is a well-established physiological phenomenon that glomerular filtration rate declines with age, typically decreasing at a rate of approximately 0.8 ml/min per year from the age of 40 onwards [9]; consequently, the older adult population is at high risk of chronic kidney disease. Statistics show that the prevalence of chronic kidney disease among the elderly is more than three times that of adolescents, whilst the relative risk of developing kidney damage is as high as 329 times that of the general adult population [6]. Of particular concern is the fact that chronic kidney disease is also associated with a significantly increased risk of dementia, disability and mortality among older adults [10–14]. There is significant heterogeneity in the health status of older adults, and the ageing process exhibits non-linear characteristics. Although the notion that ‘70 is the new 60’ reflects an overall improvement in the health status of younger older adults [15], it also implies that the burden of disease may become more concentrated and pronounced in later life (typically referring to those aged 75 and over). In light of this, authoritative bodies such as the World Health Organization (WHO) have progressively adopted higher age thresholds in their reports to identify sub-groups of older adults requiring particular attention, as this group is more susceptible to disease and faces a significantly increased risk of adverse health outcomes [16–20]. It is worth noting that the health and economic burden of chronic kidney disease on patients, families and society within this group also tends to increase with age.

Although the Global Burden of Disease (GBD) studies have extensively described the epidemiological patterns of chronic kidney disease (CKD), there remains a lack of in-depth analysis regarding the evolution of the disease burden and its driving factors among the very elderly (aged ≥ 75 years) [13,21–24]. Given that renal function declines physiologically with age, and that the very elderly have high rates of comorbidity and significant heterogeneity in treatment response, the epidemiological characteristics, disease progression and health outcomes of CKD in this population may differ fundamentally from those in younger age groups. By conducting a dedicated analysis of the GBD 2021 data for individuals aged 75 years and older, this study aims to reveal the independent impact of extreme ageing on the burden of CKD and to assess differences in the distribution of CKD risk among the very elderly across varying levels of socio-economic development. This will provide an age-specific evidence base for the formulation of targeted strategies to protect renal health in older adults, rather than a simple replication of trends observed in the general population.

## Methods

This study was reported in accordance with the STROBE (Standard for Reporting Observational Studies in Epidemiology) guidelines [25] and was approved by the Ethics Committee of the Center for Health Sciences at Peking University. As the study involved a secondary analysis of the publicly available, de-identified GBD database and did not involve any personally identifiable information, the Ethics Committee granted an exemption from informed consent. With advances in public health and increased life expectancy, the traditional age threshold of 65 years is no longer sufficient to distinguish health heterogeneity within the population [26–28]. To focus on the subgroup of older adults with the highest disease burden and most significant health vulnerabilities, this study explicitly defines the study population as older adults aged 75 years and above; this threshold aligns with the definition of ‘older adults’ used by organizations such as the World Health Organization.

### Definition of the disease and data sources

This study adopts the GBD 2021 definition of chronic kidney disease (CKD): based on an estimated glomerular filtration rate (eGFR < 60 ml/min/1.73 m²) or a urine albumin-to-creatinine ratio (> 30 mg/g), and includes patients with end-stage renal disease undergoing dialysis and those who have undergone kidney transplantation [29]. To comprehensively reflect the disease spectrum, this study covers all-cause CKD, including types caused by diabetes (type 1 and type 2), hypertension, glomerulonephritis and other causes [30], and is staged according to glomerular filtration rate (G1–G5) and urinary albumin levels (A1–A3) [31].

The study data are sourced from the Global Burden of Disease Study 2021 (GBD 2021). Using the Global Health Data Exchange query tool [32,33], we extracted data on the prevalence, incidence, mortality and disability-adjusted life years (DALYs) of CKD among people aged 75 years and older across 204 countries and regions worldwide from 1990 to 2021 [34,35]. For in-depth analysis, the study population was further subdivided into five age subgroups: 75–79, 80–84, 85–89, 90–94 and 95 years and older, and countries/regions were categorized into 21 regions based on socio-economic and geographical characteristics for analysis.

### GBD data estimation methodology and indicator definitions

The GBD database integrates over 100,000 data sources worldwide and estimates various disease burden indicators through a standardized modelling process. DALYs are the sum of years of life lost due to disability (YLDs) and years of life lost due to premature death (YLLs) [35–37]. The definitions of the key indicators used in this study are as follows [37]:

**Prevalence**: Refers to the total number of CKD cases in the population aged 75 years and over at a specific point in time (point prevalence).

**Incidence**: Refers to the number of new CKD cases in this population within a specific year, typically estimated by dividing the annual number of new cases by the population size during that year.

**Mortality**: Refers to the proportion of deaths in this population attributable to CKD relative to the total population.

**Disability-adjusted life years (DALYs)**: Represents the total loss of healthy life years due to CKD, aiding in the assessment of the overall disease burden at regional or global levels.

#### Sociodemographic Index.

Sociodemographic Index (SDI) is a comprehensive indicator of the development of a country or region, calculated from data such as the fertility rate of women under 25 years of age, the level of education per capita and the per capita income of a country. SDI takes on values ranging from 0 to 1, with the higher the value, the higher the level of socio-economic development of the region. Previous studies have shown that the SDI is closely related to disease incidence and mortality [13], so in this study, countries and regions were classified into five levels of SDI (low, low-middle, middle, high-medium, high) to investigate the relationship between the burden of CKD and SDI in the older adults.

### Statistical analysis

Data analysis for this study was carried out using R software (version 4.4.0).

**Age-standardized rates:** All rates reported in this study (prevalence, incidence, mortality and DALYs) are age-standardized rates. Although the study population was restricted to those aged 75 years and over, to account for differences in the demographic structure of the five age subgroups within this group (75–79, 80–84, 85–89, 90–94, 95 + years) and to ensure comparability across regions and over time, we age-standardized the rates using the World Standard Population. All standardized rate data and corresponding 95% uncertainty intervals (UI) are derived directly from the output of the GBD 2021 database.

**Specifications of the regression model estimated using EAPCs:** The estimated annual percentage change (EAPC) is used to measure the average trend in age-standardized rates over time. We employed a standard log-linear regression model for estimation, with the following formula: ln (ASR) = α + β × Calendar Year + ε. Here, ASR represents the age-standardized rate, Calendar Year denotes the year (1990–2021), and β is the regression coefficient. The formula for the EAPC and its 95% UI is: EAPC = (e^β – 1) × 100%. This analysis was conducted separately for different subgroups, including gender, SDI level and geographical region.

**Assumption of a linear trend:** In order to ensure the comparability of trend estimates among 204 countries/regions, the study applied the above-mentioned log-linear model to all countries uniformly, without conducting separate non-linear trend tests (such as Joinpoint analysis) for each country. We acknowledge that although logarithmic transformation handles common patterns of change in epidemiological data well and linear approximations over long time spans (32 years) are widely used, this unifying assumption may not capture the specific trend turning points that exist in individual countries. We have addressed this as a methodological limitation in the Discussion section.

**Incorporation of statistical uncertainty:** The 95% uncertainty intervals for all disease burden indicators in the analysis are derived directly from the official modelling results of GBD 2021. These intervals were generated using complex models, such as Bayesian meta-regression and Gaussian process regression, which comprehensively account for variability from multiple sources, including sampling error and uncertainty in model parameters. In this study, the 95% UI for the EAPC was calculated by applying the aforementioned log-linear model to the rate point estimates provided by GBD and the upper and lower limits of their 95% UIs, respectively. The criteria for trend interpretation were as follows: if both the EAPC estimate and the lower limit of its 95% UI were greater than 0, the trend was classified as increasing; if both the estimate and the upper limit of its 95% UI were less than 0, the trend was classified as decreasing; otherwise, the trend was considered stable [36].

Furthermore, we compared differences in disease burden across different age groups, genders, regions and SDI levels, and analyzed the burden of CKD attributable to different aetiologies. All P-values were calculated using two-sided tests, and P < 0.05 was considered statistically significant.

## Results

### CKD in older adults: Global Trends

#### Global burden and temporal changes.

From 1990 to 2021, a total of 288,520,255 (95% UI: 288,514,827–288,523,786) individuals aged 75 years and older were included in the analysis. The global epidemiology of CKD in this population showed divergent trends across different metrics. While the number of prevalent cases increased by 140.33% to 126,963,771 (95% UI: 116,043,499–137,447,863) in 2021, the age-standardized prevalence rate exhibited a slight decline, from 4,502.1 (95% UI: 4,103.5–4,911.2) to 4,400.4 (95% UI: 4,021.9–4,763.7) per 100,000 population, corresponding to an estimated annual percentage change (EAPC) of −0.06% (95% UI: −0.09% – −0.03%).

In contrast, the age-standardized incidence, mortality, and DALYs rates all demonstrated significant increases. The incidence rate rose from 1.55% to 1.82% (EAPC = 0.50%; 95% UI: 0.47–0.53). The mortality rate increased from 160.55 (95% UI: 145.34–178.01) to 246.90 (95% UI: 213.17–267.13) per 100,000 (EAPC = 1.55%; 95% UI: 1.48–1.61). Similarly, the DALYs rate showed substantial growth (EAPC = 1.07%; 95% UI: 1.02–1.12).

#### Age and sex patterns.

A critical divergence was observed in age-specific trends. The EAPC for incidence decreased progressively with advancing age, from 0.66% (95% UI: 0.62%–0.70%) in the 75–79 years group to 0.10% (95% UI: 0.02%–0.19%) in the 90–94 years group. Conversely, the EAPC for mortality showed a marked, graded increase with age, from 0.79% (95% UI: 0.75%–0.82%) in the 75–79 years group to 2.29% (95% UI: 2.13%–2.45%) in those aged ≥ 95 years.

**Fig 1:**
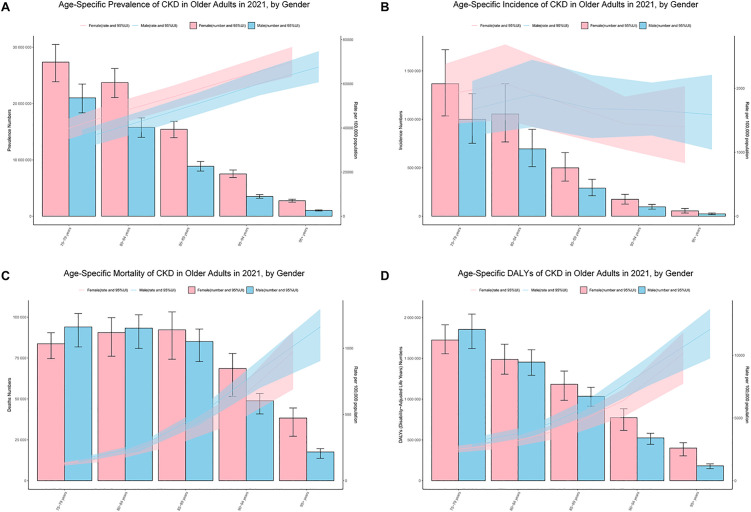
Age-Specific differences of CKD in the older adults in 2021, by Gender (A) prevalence (B) incidence (C) mortality (D) DALYs.

Sex disparities were also evident. In 2021, women had higher prevalence and incidence rates across all age groups. However, men bore a higher burden of mortality and DALYs. Notably, the rate of increase in mortality (EAPC) was steeper for women than for men in every age stratum (Fig 1).

### CKD in older adults: SDI regional trends

The burden of CKD among older adults varied markedly across SDI regions, with distinct patterns in static levels and temporal trends. In 2021, the high-SDI region carried the largest absolute burden, accounting for the most prevalent cases (44.2 million), incident cases (2.35 million), deaths (246,298), and DALYs (3.35 million).

#### Divergent trends in prevalence and mortality.

A key observation was the contrasting relationship between SDI and trends in prevalence versus mortality. The age-standardized prevalence rate showed a slight increase in high-SDI regions (EAPC = 0.07%; 95% UI: 0.03%–0.11%) but significant decreases in high-middle and middle SDI regions (EAPC = −0.21%; 95% UI: −0.27% – −0.14% and EAPC = −0.08%; 95% UI: −0.13% – −0.03%, respectively), while remaining relatively stable in low and low-middle SDI regions.

**Fig 2:**
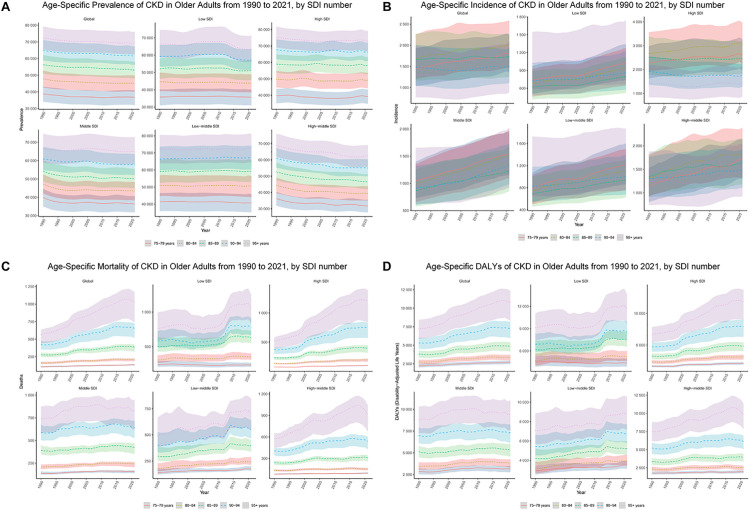
Age-Specific differences of CKD in the older adults from 1990 to 2021, by SDI number (A) prevalence (B) incidence (C) mortality (D) DALYs.

In stark contrast, the mortality rate presented a more complex picture. Throughout the study period, the low-SDI region consistently had the highest mortality rate (336.51, 95% UI: 300.78–382.43, per 100,000 in 2021). However, the most rapid annual increase in mortality occurred in the high-SDI region (EAPC = 2.56%; 95% UI: 2.42%–2.71%), far exceeding the growth in low-SDI regions (EAPC = 0.41%; 95% UI: 0.30%–0.53%) (Fig 2C). Consequently, the high-SDI region’s mortality ranking rose from fourth highest in 1990 to second highest in 2021.

#### Incidence and DALYs: growth and inequality.

Trends in incidence and DALYs further illustrated the heterogeneity. The greatest increase in the incidence rate was observed in the middle-SDI region (EAPC = 1.26%; 95% UI: 1.23%–1.28%), while the increase was most modest in the high-SDI region (EAPC = 0.26%; 95% UI: 0.21%–0.30%) (Fig 2B). The number of incident cases in the low-middle SDI region surged by 313% from 1990 to 2021.

Similarly, the number of CKD-associated DALYs increased dramatically in all regions, with the most substantial relative increase (227%) and the highest final count in the high-SDI region. The low-SDI region had the smallest increase in the DALYs rate (EAPC = 0.22%; 95% UI: 0.14%–0.30%) (Fig 2D).

### CKD in older adults: geographic regional and national-level trends

#### Geographic regional of disease burden.

The burden of CKD among older adults exhibited profound geographic disparities. In 2021, the highest age-standardized rates were concentrated in different regions: Central Asia had the highest prevalence rate, while North Africa and the Middle East had the highest mortality rate, and Central Sub-Saharan Africa had the highest DALYs rate. This pattern aligns with the finding that the heaviest burden often falls on low- and middle-SDI regions.

**Fig 3:**
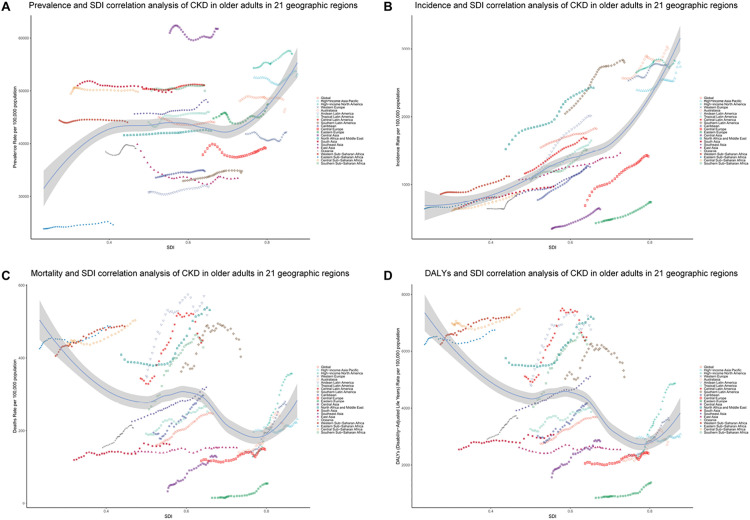
Correlation analysis of CKD in the older adults, by 21 geographic regions (A) prevalence (B) incidence (C) mortality (D) DALYs.

Temporal trends (1990–2021) also varied markedly. While the age-standardized incidence of CKD increased in all 21 GBD regions, the growth was most rapid in Andean Latin America (EAPC = 2.34%; 95% UI: 2.20%–2.48%) and slowest in High-income North America (EAPC = 0.16%; 95% UI: 0.10%–0.22%). Mortality rates increased universally, with the steepest rise observed in Eastern Europe (EAPC = 4.49%; 95% UI: 3.84%–5.14%) and the most modest in Eastern Sub-Saharan Africa (EAPC = 0.27%; 95% UI: 0.20%–0.35%). In 2021, a majority of regions (13/21) had mortality rates above the global average (Fig 3).

#### National-Level burden and inequality.

**Fig 4:**
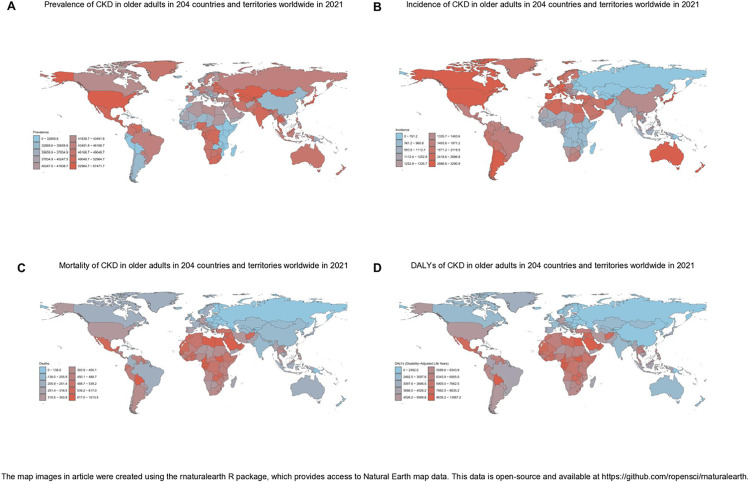
GBD 2021 data on CKD among older adults in 204 countries (A) prevalence (B) incidence (C) mortality (D) DALYs.

Analysis across 204 countries and territories revealed even starker inequalities. The People’s Republic of China contributed the largest number of prevalent cases, incident cases, deaths, and DALYs globally in 2021, reflecting its large aging population. Critically, the distribution of the mortality burden was highly skewed. In 2021, the vast majority of countries (169 out of 204, 82.8%) had age-standardized mortality rates above the global average (246.90, 95% UI: 213.17–267.13, per 100,000), underscoring the pervasiveness of elevated CKD-related death risk in the older adult population worldwide (Fig 4).

## Discussion

This study provides a comprehensive analysis of the global burden of chronic kidney disease (CKD) from 1990 to 2021, specifically focusing on adults aged 75 years and older. We found that while the age-standardized prevalence rate remained relatively stable, the incidence, mortality, and disability-adjusted life years (DALYs) rates increased significantly. These burdens exhibited substantial heterogeneity across ages, sexes, socio-demographic levels, and geographic regions. Our findings confirm and extend prior knowledge by delineating the unique epidemiological profile of CKD in the fastest-growing segment of the global population—the oldest-old [38,39].

The observed divergence between stable prevalence and rising mortality presents a “high-age CKD paradox” in this population. CKD is a disease with diverse aetiologies influenced by genetic, metabolic, and environmental factors, whose precise mechanisms in older adults require further exploration [38]. The marginal decline in prevalence may reflect advancements in managing hypertension and cardiovascular comorbidities over the past decades. However, the marked rise in incidence could be driven by the increasing prevalence of key risk factors like diabetes and obesity in aging societies, coupled with improved detection. The steep acceleration in mortality, particularly with advancing age, underscores that extreme age itself is a critical amplifier of risk for fatal outcomes. This is likely multifactorial, involving cumulative renal function decline, heightened vulnerability to acute kidney injury, and the blunted physiological response to disease and treatment commonly seen in advanced age [39].

Our analysis reveals a complex relationship between socio-demographic development and CKD burden. Consistent with some studies, we found a negative correlation between DALYs levels and SDI (Spearman r = −0.47; p < 0.001). This may be partly explained by challenges in low-SDI regions, where non-specific symptoms of CKD (e.g., dizziness, weakness) often lead to delayed diagnosis [40–42]. Failure to identify and treat high-risk patients in a timely manner likely increases progression to end-stage renal disease, elevating both premature death and disability, thereby contributing to higher DALYs[40–42].

However, we also observed a “high-SDI region paradox”: although low-SDI regions carried the highest mortality rates throughout the study period, the fastest growth in mortality and DALYs occurred in high-SDI regions. This counterintuitive finding may be primarily related to the more advanced degree of population ageing in high-SDI areas [43]. The proportion and absolute number of the “oldest-old” are greater in these regions, which likely drives a rapid increase in the number of individuals at the highest risk for CKD-related death. Furthermore, the strong positive correlation between SDI and the EAPC of incidence (Spearman r = 0.64; p < 0.0001) may not solely indicate a higher risk of developing CKD. Instead, it could reflect more intensive diagnostic efforts and better case ascertainment in well-resourced health systems, alongside a higher prevalence of lifestyle risk factors such as diabetes and hypertension. Diabetes mellitus, a major driver of CKD [11,44,45], is more prevalent in high-SDI settings, and its coexistence with an aging population may compound the increase in CKD incidence and severe outcomes [46–48].

### Limitations

This study has several limitations. Firstly, our research and analysis are based on the GBD database, which incorporates data from various countries. Consequently, the accuracy and availability of the data may be influenced by the quality of national registry data, potentially leading to selection bias and underrepresentation of certain demographic groups in some regions. Secondly, variations in the quality and completeness of national registry data may result in underreporting and missed cases of CKD among older adults. Thirdly, a large number of older adults with CKD remain undiagnosed, and data on risk factors specific to this population (such as polypharmacy and comorbidity) are relatively limited in the GBD, which restricts in-depth exploration of the aetiology and natural history of the disease. Fourthly, the GBD database classifies the aetiology of CKD under the broad category of ‘other and unspecified causes’, which may account for a significant proportion of cases. This broad categorization may obscure the specific distribution of aetiological factors, thereby limiting research into disease mechanisms and targeted prevention strategies. Fifth, to better investigate the impact of age on elderly CKD, this study selected participants aged 75 years and older as subjects. This approach may have overlooked trends and risk factors potentially influencing the progression of elderly CKD in younger age groups. Sixth, in the trend analysis, to maintain cross-national comparability, we uniformly applied a log-linear model to estimate annual percentage changes for all countries. This method assumes that all regions maintained a constant log-linear trend between 1990 and 2021. Whilst this facilitates comparison, it may fail to identify non-linear inflection points in certain countries or periods (such as trend shifts following policy interventions), thereby simplifying dynamic processes that may in reality be more complex.

## Conclusions

Based on the Global Burden of Disease, Injuries, and Risk Factors Study (GBD) 2021, this study presents a comprehensive, longitudinal assessment (1990–2021) of the global burden of chronic kidney disease (CKD) among adults aged ≥75 years. Our analysis reveals a critical shift in the epidemiological profile of CKD within this rapidly growing demographic: the burden is evolving from a state of high prevalence toward one characterized by elevated incidence, accelerated mortality, and substantial health loss. A notable paradox emerges—a stable or even slightly declining prevalence coexists with sharply rising mortality—highlighting how extreme population aging intensifies disease progression and exacerbates the risk of adverse outcomes, thereby representing a major and growing public health challenge.

The distribution of this burden is markedly heterogeneous and closely tied to levels of socio-demographic development. Low- and middle-income regions continue to carry the heaviest absolute burden of CKD-related deaths and healthy life years lost, largely attributable to systemic gaps in healthcare access, delayed diagnosis, and suboptimal disease management. In these settings, strengthening foundational kidney health systems and advancing universal health coverage remain urgent priorities to reduce preventable morbidity and mortality. In contrast, high SDI regions—despite superior healthcare infrastructure and diagnostic capacity—are experiencing the most rapid increase in CKD-related mortality. This pattern signals an epidemiological transition wherein the principal challenge is shifting from “high mortality due to untreated disease” to “high morbidity and mortality driven by advanced population aging, intensive case finding, and the rising prevalence of lifestyle-related risk factors.”

In summary, the global burden of CKD in older adults is not only increasing in magnitude but also growing in complexity. Effectively addressing this dual challenge necessitates a move away from uniform, one-size-fits-all approaches toward the design and implementation of stratified, context-specific strategies for prevention, screening, management, and long-term care. Such strategies must be carefully tailored to the distinct epidemiological patterns and demographic realities observed across different geographic regions and age strata within the older population. By delineating the evolving landscape of CKD in the oldest-old worldwide, this study provides a robust, evidence-based foundation to inform health policy formulation, guide optimal resource allocation, and steer future research aimed at mitigating the growing burden of CKD in an aging global population.

## Supporting information

S1 Table GBD 2021 prevalence data tables (Global, 5SDI, 21 Regions). (DOCX)

S2 Table GBD 2021 incidence data tables (Global, 5SDI, 21 Regions). (DOCX)

S3 Table GBD 2021 mortality data tables (Global, 5SDI, 21 Regions). (DOCX)

S4 Table GBD 2021 DALYs data tables (Global, 5SDI, 21 Regions). (DOCX)
