# Supplementary material for: Global, Regional, and National Burden of chronic kidney disease in older adults from 1990 to 2021: Results from the Global Burden of Disease Study 2021
Source: PLoS One. 2026 Jul 31;21(7):e0354811. doi: 10.1371/journal.pone.0354811 (PMC13426924; doi:10.1371/journal.pone.0354811)
Supplement: S1 Table — (DOCX) [file pone.0354811.s001.docx]

| **S1 Table. GBD 2021 prevalence data tables (Global, 5SDI, 21 Regions)** | | | | | | | |
| --- | --- | --- | --- | --- | --- | --- | --- |
| Location | | Rate per 100,000 (95% UI) | |  |  |  |  |
|  |  | 1990 |  | 2021 |  | 1990-2021 | |
|  |  | Prevalent cases | Prevalent rate | Prevalent cases | Prevalent rate | Cases change | EAPCs |
| Global | | 52,829,922.87(48,153,123.86-57,630,360.87) | 45,020.72(41,035.24-49,111.57) | 126,963,770.53(116,043,498.72-137,447,863.12) | 44,003.83(40,219.02-47,637.46) | 1.40(1.36-1.45) | -0.06(-0.09--0.03) |
| SDI | |  |  |  |  |  |  |
|  | High | 20,901,247.03(19,180,439.69-22,683,510.67) | 47,494.72(43,584.46-51,544.62) | 44,182,412.90(40,748,341.24-47,588,261.63) | 49,026.00(45,215.46-52,805.22) | 1.11(1.07-1.17) | 0.07(0.03-0.11) |
|  | High middle | 12,828,652.64(11,665,047.28-14,049,702.38) | 41,636.29(37,859.73-45,599.29) | 26,471,630.57(24,152,248.72-28,625,415.28) | 38,614.78(35,231.45-41,756.56) | 1.06(1.01-1.12) | -0.21(-0.27--0.14) |
|  | Middle | 10,581,513.82(9,602,723.77-11,617,302.52) | 44,388.67(40,282.72-48,733.73) | 33,193,295.09(30,180,243.90-36,169,721.33) | 41,823.21(38,026.80-45,573.48) | 2.14(2.07-2.21) | -0.08(-0.13--0.03) |
|  | Low middle | 6,591,298.92(5,918,910.10-7,251,241.12) | 47,292.59(42,468.19-52,027.67) | 18,310,660.13(16,495,229.05-19,953,339.84) | 47,377.62(42,680.32-51,627.95) | 1.78(1.73-1.83) | 0.00(-0.01-0.01) |
|  | Low | 1,871,341.16(1,676,981.78-2,056,857.87) | 40,641.62(36,420.54-44,670.65) | 4,696,791.89(4,234,732.87-5,135,938.26) | 40,617.59(36,621.73-44,415.30) | 1.51(1.47-1.55) | 0.02(-0.01-0.04) |
| Regions | |  |  |  |  |  |  |
|  | Andean Latin America | 186,357.16(169,300.95-203,112.01) | 30,740.93(27,927.39-33,504.76) | 632,838.02(576,932.56-687,348.69) | 31,838.98(29,026.29-34,581.48) | 2.40(2.23-2.55) | 0.15(0.12-0.19) |
|  | Australasia | 423,195.97(389,683.22-454,662.55) | 48,084.24(44,276.47-51,659.53) | 1,051,299.80(967,942.61-1,152,643.79) | 46,497.84(42,811.04-50,980.17) | 1.48(1.32-1.65) | -0.06(-0.11--0.01) |
|  | Caribbean | 291,458.84(263,787.46-318,915.18) | 33,548.67(30,363.53-36,709.06) | 684,697.68(630,419.54-741,035.14) | 34,767.67(32,011.53-37,628.39) | 1.35(1.25-1.47) | 0.13(0.12-0.14) |
|  | Central Asia | 889,583.84(817,416.49-962,595.40) | 60,982.87(56,035.64-65,987.97) | 1,196,821.90(1,094,223.77-1,293,894.77) | 61,759.94(56,465.54-66,769.22) | 0.35(0.30-0.39) | -0.07(-0.12--0.01) |
|  | Central Europe | 1,938,693.95(1,759,784.88-2,113,151.37) | 37,893.76(34,396.80-41,303.71) | 3,478,476.06(3,224,114.76-3,728,654.58) | 39,128.74(36,267.47-41,942.95) | 0.79(0.73-0.87) | -0.01(-0.08-0.07) |
|  | Central Latin America | 1,186,141.85(1,083,042.92-1,297,139.97) | 50,369.16(45,991.10-55,082.66) | 4,167,446.62(3,852,507.98-4,500,460.32) | 51,067.44(47,208.22-55,148.15) | 2.51(2.39-2.66) | 0.08(0.06-0.09) |
|  | Central Sub-Saharan Africa | 173,129.97(156,455.96-189,865.18) | 49,437.87(44,676.55-54,216.67) | 479,274.31(440,491.24-516,474.22) | 49,828.40(45,796.27-53,695.94) | 1.77(1.67-1.87) | -0.02(-0.04-0.00) |
|  | East Asia | 7,752,761.70(6,944,204.94-8,620,436.23) | 39,873.79(35,715.25-44,336.39) | 22,946,864.99(20,793,800.46-25,104,321.96) | 33,447.98(30,309.61-36,592.75) | 1.96(1.86-2.06) | -0.36(-0.50--0.22) |
|  | Eastern Europe | 4,391,904.07(3,978,588.91-4,822,750.36) | 44,773.67(40,560.09-49,165.97) | 5,775,278.08(5,249,211.11-6,293,260.35) | 47,467.03(43,143.28-51,724.32) | 0.31(0.28-0.36) | 0.07(-0.01-0.14) |
|  | Eastern Sub-Saharan Africa | 359,569.74(317,090.81-400,820.84) | 23,853.67(21,035.64-26,590.25) | 902,636.08(805,778.31-997,836.03) | 24,621.62(21,979.58-27,218.43) | 1.51(1.46-1.56) | 0.17(0.15-0.18) |
|  | High-income Asia Pacific | 3,550,667.96(3,225,247.28-3,871,510.35) | 52,524.61(47,710.70-57,270.79) | 12,151,221.13(11,198,019.60-13,090,086.70) | 53,154.34(48,984.65-57,261.32) | 2.42(2.33-2.53) | 0.07(0.02-0.12) |
|  | High-income North America | 7,798,144.94(7,140,662.84-8,513,426.15) | 54,332.11(49,751.22-59,315.69) | 14,786,076.84(13,524,998.99-16,099,340.24) | 56,982.99(52,123.02-62,044.08) | 0.90(0.86-0.94) | 0.20(0.18-0.22) |
|  | North Africa and Middle East | 1,578,971.91(1,417,312.96-1,741,403.30) | 41,602.85(37,343.45-45,882.60) | 4,772,925.42(4,328,947.54-5,210,534.97) | 42,438.79(38,491.13-46,329.82) | 2.02(1.95-2.09) | 0.08(0.07-0.08) |
|  | Oceania | 18,002.24(16,227.79-19,734.11) | 38,423.27(34,635.96-42,119.71) | 54,693.59(49,577.17-59,900.19) | 39,112.80(35,453.92-42,836.18) | 2.04(1.90-2.15) | 0.17(0.13-0.21) |
|  | South Asia | 6,273,373.08(5,596,419.58-6,920,552.53) | 50,998.70(45,495.48-56,259.87) | 19,876,534.65(17,808,799.53-21,760,386.27) | 49,768.02(44,590.70-54,484.91) | 2.17(2.12-2.23) | -0.11(-0.13--0.09) |
|  | Southeast Asia | 2,689,663.23(2,421,321.72-2,947,588.45) | 45,625.08(41,073.17-50,000.29) | 7,895,585.63(7,177,424.67-8,576,354.98) | 47,858.53(43,505.45-51,984.97) | 1.94(1.87-2.01) | 0.16(0.12-0.19) |
|  | Southern Latin America | 507,960.29(458,918.66-554,604.67) | 32,924.91(29,746.14-35,948.30) | 1,186,056.87(1,085,915.31-1,301,412.71) | 34,789.00(31,851.68-38,172.58) | 1.33(1.18-1.54) | 0.21(0.19-0.23) |
|  | Southern Sub-Saharan Africa | 351,353.98(320,657.36-383,596.50) | 50,055.60(45,682.41-54,649.02) | 720,181.81(655,357.34-785,244.47) | 50,000.96(45,500.31-54,518.15) | 1.05(1.01-1.10) | 0.05(0.03-0.07) |
|  | Tropical Latin America | 1,067,126.32(964,673.63-1,172,309.43) | 44,060.09(39,829.96-48,402.94) | 3,908,164.96(3,564,375.58-4,254,906.40) | 45,988.03(41,942.60-50,068.19) | 2.66(2.58-2.75) | 0.19(0.15-0.23) |
|  | Western Europe | 10,467,445.02(9,574,146.78-11,368,464.18) | 41,820.21(38,251.25-45,420.02) | 18,345,771.50(16,911,427.63-19,684,135.67) | 42,161.40(38,865.06-45,237.17) | 0.75(0.69-0.83) | -0.04(-0.10-0.01) |
|  | Western Sub-Saharan Africa | 934,416.82(848,781.90-1,020,450.30) | 44,422.03(40,350.96-48,512.05) | 1,950,924.61(1,775,776.16-2,121,761.68) | 44,028.06(40,075.35-47,883.48) | 1.09(1.06-1.12) | 0.02(0.00-0.04) |
| Prevalence of CKD in the older adults Between 1,990 and 2,19 at the Global and Regional Level. EAPC, estimated annual percentage change; SDI, Sociodemographic Index; UI, uncertainty interval. EAPC is expressed as 95% UIs. | | | | | | | |
