# Supplementary material for: Global, Regional, and National Burden of chronic kidney disease in older adults from 1990 to 2021: Results from the Global Burden of Disease Study 2021
Source: PLoS One. 2026 Jul 31;21(7):e0354811. doi: 10.1371/journal.pone.0354811 (PMC13426924; doi:10.1371/journal.pone.0354811)
Supplement: S2 Table — (DOCX) [file pone.0354811.s002.docx]

| **S2 Table. GBD 2021 incidence data tables (Global, 5SDI, 21 Regions)** | | | | | | | |
| --- | --- | --- | --- | --- | --- | --- | --- |
| Location | | Rate per 100 000 (95%UI) | | | | |  |
|  |  | 1990 |  | 2021 |  | 1990-2021 | |
|  |  | Incident cases | Incidence rate | Incident cases | Incidence rate | Cases change | EAPCs |
| Global | | 1,813,315.55(1,456,822.09-2,216,298.66) | 1,545.28(1,241.48-1,888.69) | 5,257,946.27(4,345,554.69-6,161,082.03) | 1,822.33(1,506.11-2,135.34) | 1.90(1.62-2.30) | 0.50(0.47-0.53) |
| SDI | |  |  |  |  |  |  |
|  | High | 1,056,317.86(859,960.01-1,273,267.23) | 2,400.31(1,954.12-2,893.29) | 2,353,387.00(1,981,812.70-2,700,181.36) | 2,611.38(2,199.07-2,996.19) | 1.23(1.01-1.53) | 0.26(0.21-0.30) |
|  | High middle | 377,326.40(300,742.00-466,174.74) | 1,224.64(976.08-1,513.00) | 1,176,198.18(974,847.21-1,372,594.04) | 1,715.75(1,422.03-2,002.23) | 2.12(1.76-2.62) | 1.01(0.95-1.08) |
|  | Middle | 239,262.81(181,202.77-307,978.62) | 1,003.69(760.13-1,291.95) | 1,171,800.67(936,690.08-1,400,533.65) | 1,476.46(1,180.22-1,764.66) | 3.90(3.23-4.93) | 1.26(1.23-1.28) |
|  | Low middle | 106,445.01(81,613.02-136,818.47) | 763.74(585.57-981.67) | 440,021.52(337,557.94-547,439.93) | 1,138.53(873.41-1,416.46) | 3.13(2.77-3.63) | 1.20(1.13-1.27) |
|  | Low | 32,511.03(24,924.91-41,327.57) | 706.07(541.32-897.55) | 112,172.62(85,874.28-140,057.02) | 970.06(742.64-1,211.21) | 2.45(2.13-2.86) | 0.98(0.89-1.07) |
| Regions | |  |  |  |  |  |  |
|  | Andean Latin America | 6,429.93(5,093.05-8,164.84) | 1,060.66(840.13-1,346.85) | 40,013.68(31,888.89-48,749.50) | 2,013.15(1,604.37-2,452.66) | 5.22(4.31-6.22) | 2.34(2.20-2.48) |
|  | Australasia | 22,612.06(18,987.28-26,844.42) | 2,569.22(2,157.37-3,050.11) | 68,016.22(58,011.60-78,643.21) | 3,008.28(2,565.79-3,478.30) | 2.01(1.60-2.53) | 0.51(0.42-0.60) |
|  | Caribbean | 6,710.56(5,030.28-8,638.55) | 772.43(579.02-994.35) | 25,142.55(19,077.35-30,668.91) | 1,276.69(968.71-1,557.31) | 2.75(2.28-3.44) | 1.67(1.61-1.73) |
|  | Central Asia | 5,068.20(3,681.00-6,818.61) | 347.44(252.34-467.43) | 12,312.25(9,073.77-15,923.08) | 635.35(468.24-821.68) | 1.43(1.19-1.71) | 2.26(2.11-2.41) |
|  | Central Europe | 35,299.09(26,366.43-46,281.60) | 689.96(515.36-904.62) | 125,920.64(99,525.88-154,041.71) | 1,416.46(1,119.55-1,732.79) | 2.57(2.09-3.29) | 1.99(1.78-2.20) |
|  | Central Latin America | 27,399.48(20,305.40-35,569.66) | 1,163.51(862.26-1,510.46) | 136,630.63(106,057.49-163,217.91) | 1,674.26(1,299.62-2,000.05) | 3.99(3.22-5.03) | 1.29(1.23-1.35) |
|  | Central Sub-Saharan Africa | 2,153.78(1,654.53-2,749.15) | 615.02(472.46-785.03) | 8,457.62(6,597.57-10,419.70) | 879.31(685.93-1,083.30) | 2.93(2.44-3.59) | 1.09(0.94-1.25) |
|  | East Asia | 208,152.40(155,985.33-265,104.58) | 1,070.56(802.26-1,363.48) | 1,005,180.60(822,168.84-1,169,554.86) | 1,465.18(1,198.42-1,704.78) | 3.83(3.07-4.99) | 1.01(0.97-1.05) |
|  | Eastern Europe | 42,356.94(31,016.78-56,592.94) | 431.81(316.20-576.94) | 89,768.29(67,136.05-113,773.41) | 737.81(551.79-935.10) | 1.12(0.83-1.52) | 1.80(1.67-1.94) |
|  | Eastern Sub-Saharan Africa | 9,695.64(7,432.97-12,213.65) | 643.20(493.10-810.25) | 30,167.40(23,483.53-37,188.71) | 822.89(640.57-1,014.41) | 2.11(1.77-2.56) | 0.71(0.59-0.82) |
|  | High-income Asia Pacific | 161,807.99(128,749.64-196,107.72) | 2,393.61(1,904.58-2,901.00) | 625,651.65(531,984.09-718,321.94) | 2,736.85(2,327.11-3,142.23) | 2.87(2.47-3.42) | 0.45(0.37-0.53) |
|  | High-income North America | 380,441.47(308,052.69-461,821.64) | 2,650.65(2,146.30-3,217.66) | 731,821.88(603,499.20-854,473.98) | 2,820.32(2,325.78-3,293.00) | 0.92(0.72-1.19) | 0.16(0.10-0.22) |
|  | North Africa and Middle East | 57,814.95(44,887.55-72,738.01) | 1,523.31(1,182.70-1,916.51) | 272,912.36(215,609.78-335,876.32) | 2,426.62(1,917.11-2,986.47) | 3.72(3.21-4.35) | 1.43(1.33-1.52) |
|  | Oceania | 301.82(223.72-393.89) | 644.20(477.51-840.70) | 1,201.57(923.23-1,502.96) | 859.27(660.23-1,074.81) | 2.98(2.45-3.59) | 1.04(0.87-1.22) |
|  | South Asia | 80,373.17(60,416.82-104,617.45) | 653.38(491.15-850.48) | 387,800.39(294,904.65-490,325.31) | 971.00(738.40-1,227.70) | 3.82(3.39-4.38) | 1.20(1.11-1.28) |
|  | Southeast Asia | 49,098.39(37,591.83-62,616.78) | 832.86(637.67-1,062.18) | 207,794.82(159,131.20-258,470.43) | 1,259.53(964.56-1,566.70) | 3.23(2.76-3.88) | 1.29(1.20-1.38) |
|  | Southern Latin America | 31,559.05(25,370.86-38,861.59) | 2,045.59(1,644.49-2,518.93) | 95,508.95(76,936.43-110,338.28) | 2,801.43(2,256.67-3,236.40) | 2.03(1.53-2.62) | 1.11(1.00-1.23) |
|  | Southern Sub-Saharan Africa | 7,856.63(6,111.56-9,875.49) | 1,119.29(870.68-1,406.91) | 21,818.93(17,006.56-26,842.37) | 1,514.85(1,180.74-1,863.62) | 1.78(1.51-2.13) | 0.96(0.83-1.08) |
|  | Tropical Latin America | 26,157.29(19,559.51-33,468.61) | 1,080.00(807.58-1,381.87) | 140,119.34(110,349.16-165,219.85) | 1,648.81(1,298.50-1,944.17) | 4.36(3.46-5.61) | 1.41(1.35-1.47) |
|  | Western Europe | 633,982.08(523,940.62-760,887.49) | 2,532.93(2,093.28-3,039.95) | 1,181,992.47(1,013,979.57-1,340,818.31) | 2,716.40(2,330.28-3,081.41) | 0.86(0.67-1.14) | 0.31(0.25-0.38) |
|  | Western Sub-Saharan Africa | 18,044.62(13,808.84-22,638.68) | 857.84(656.47-1,076.24) | 49,714.03(38,380.67-62,684.83) | 1,121.94(866.17-1,414.66) | 1.76(1.52-2.05) | 0.83(0.71-0.95) |
| Incidence of CKD in the older adults Between 1,990 and 2,19 at the Global and Regional Level. EAPC, estimated annual percentage change; SDI, Sociodemographic Index; UI, uncertainty interval. EAPC is expressed as 95% UIs. | | | | | | | |
