# Supplementary material for: Global, Regional, and National Burden of chronic kidney disease in older adults from 1990 to 2021: Results from the Global Burden of Disease Study 2021
Source: PLoS One. 2026 Jul 31;21(7):e0354811. doi: 10.1371/journal.pone.0354811 (PMC13426924; doi:10.1371/journal.pone.0354811)
Supplement: S3 Table — (DOCX) [file pone.0354811.s003.docx]

| **S3 Table. GBD 2021 mortality data tables (Global, 5SDI, 21 Regions)** | | | | | | | |
| --- | --- | --- | --- | --- | --- | --- | --- |
| Location | | Rate per 100 000 (95% UI) | |  |  |  |  |
|  |  | 1990 |  | 2021 |  | 1990-2021 | |
|  |  | Mortality cases | Mortality rate | Mortality cases | Mortality rate | Cases change | EAPCs |
| Global | | 188,395.82(170,550.08-208,887.25) | 160.55(145.34-178.01) | 712,376.68(615,069.17-770,733.58) | 246.90(213.17-267.13) | 2.78(2.34-3.59) | 1.55(1.48-1.61) |
| SDI | |  |  |  |  |  |  |
|  | High | 61,123.05(54,113.50-64,475.74) | 138.89(122.96-146.51) | 246,297.82(199,324.88-272,798.50) | 273.30(221.18-302.70) | 3.03(2.68-3.25) | 2.56(2.42-2.71) |
|  | High middle | 39,766.45(35,779.69-43,901.84) | 129.06(116.13-142.49) | 130,549.64(111,261.28-144,979.77) | 190.44(162.30-211.49) | 2.28(1.87-2.56) | 1.30(1.23-1.38) |
|  | Middle | 48,406.78(43,249.54-54,978.66) | 203.06(181.43-230.63) | 201,249.76(171,667.97-220,615.14) | 253.57(216.30-277.97) | 3.16(2.34-3.59) | 0.87(0.80-0.93) |
|  | Low middle | 25,196.73(21,792.36-31,708.85) | 180.79(156.36-227.51) | 94,712.70(83,789.23-110,046.63) | 245.06(216.80-284.74) | 2.76(1.81-3.34) | 0.99(0.92-1.05) |
|  | Low | 13,682.48(12,053.23-16,357.36) | 297.15(261.77-355.25) | 38,911.81(34,780.37-44,222.35) | 336.51(300.78-382.43) | 1.84(1.42-2.21) | 0.41(0.30-0.53) |
| Regions | |  |  |  |  |  |  |
|  | Andean Latin America | 2,126.43(1,895.24-2,394.48) | 350.77(312.63-394.99) | 10,297.91(8,428.64-12,312.38) | 518.10(424.06-619.45) | 3.84(2.88-4.96) | 1.36(1.03-1.68) |
|  | Australasia | 1,238.38(1,101.10-1,326.07) | 140.71(125.11-150.67) | 4,798.41(3,920.51-5,306.89) | 212.23(173.40-234.72) | 2.87(2.54-3.17) | 2.05(1.75-2.36) |
|  | Caribbean | 1,691.92(1,556.17-1,889.69) | 194.75(179.12-217.52) | 5,854.34(5,025.68-6,567.99) | 297.27(255.19-333.51) | 2.46(2.05-2.87) | 1.78(1.62-1.94) |
|  | Central Asia | 487.56(377.43-696.57) | 33.42(25.87-47.75) | 2,475.79(2,201.53-2,733.77) | 127.76(113.61-141.07) | 4.08(2.43-5.67) | 4.22(3.88-4.57) |
|  | Central Europe | 5,998.94(5,623.15-6,298.68) | 117.26(109.91-123.11) | 13,214.34(11,825.30-14,305.49) | 148.65(133.02-160.92) | 1.20(1.03-1.34) | 0.91(0.74-1.08) |
|  | Central Latin America | 8,099.37(7,587.85-8,515.61) | 343.94(322.22-361.61) | 36,605.34(32,459.58-40,055.89) | 448.56(397.76-490.84) | 3.52(3.07-3.93) | 1.50(1.13-1.87) |
|  | Central Sub-Saharan Africa | 1,550.98(1,281.98-1,873.28) | 442.89(366.07-534.92) | 4,843.26(3,505.54-6,358.42) | 503.54(364.46-661.06) | 2.12(1.22-3.11) | 0.37(0.27-0.48) |
|  | East Asia | 27,680.20(24,303.08-32,789.96) | 142.36(124.99-168.64) | 105,197.91(86,133.46-124,336.21) | 153.34(125.55-181.24) | 2.80(1.92-3.59) | 0.34(0.25-0.43) |
|  | Eastern Europe | 1,520.39(1,439.04-1,566.26) | 15.50(14.67-15.97) | 6,713.58(5,963.01-7,248.10) | 55.18(49.01-59.57) | 3.42(3.06-3.71) | 4.49(3.84-5.14) |
|  | Eastern Sub-Saharan Africa | 6,414.94(5,561.51-7,874.08) | 425.56(368.95-522.36) | 17,856.08(15,651.55-20,412.30) | 487.07(426.93-556.80) | 1.78(1.24-2.23) | 0.27(0.20-0.35) |
|  | High-income Asia Pacific | 13,319.83(11,641.07-14,212.59) | 197.04(172.21-210.25) | 53,021.78(40,557.03-60,020.94) | 231.94(177.41-262.56) | 2.98(2.49-3.29) | 0.35(0.24-0.47) |
|  | High-income North America | 18,468.91(16,002.33-19,786.62) | 128.68(111.49-137.86) | 92,604.71(75,902.20-102,318.19) | 356.88(292.51-394.32) | 4.01(3.75-4.24) | 3.89(3.64-4.13) |
|  | North Africa and Middle East | 15,531.44(12,345.69-24,645.49) | 409.22(325.28-649.36) | 59,846.28(51,673.36-67,151.03) | 532.13(459.46-597.08) | 2.85(1.37-3.83) | 1.17(0.92-1.43) |
|  | Oceania | 71.92(55.03-100.42) | 153.50(117.45-214.32) | 290.36(241.01-367.19) | 207.64(172.35-262.59) | 3.04(1.82-4.66) | 1.13(1.05-1.20) |
|  | South Asia | 14,732.72(12,555.38-17,186.06) | 119.77(102.07-139.71) | 64,834.98(54,287.69-75,597.77) | 162.34(135.93-189.29) | 3.40(2.38-4.29) | 0.84(0.67-1.00) |
|  | Southeast Asia | 13,291.05(11,701.74-15,802.50) | 225.46(198.50-268.06) | 52,706.73(45,426.48-59,584.77) | 319.48(275.35-361.17) | 2.97(2.08-3.61) | 1.03(0.99-1.08) |
|  | Southern Latin America | 5,595.29(5,174.22-5,953.27) | 362.67(335.38-385.88) | 13,753.61(11,874.95-14,855.59) | 403.42(348.31-435.74) | 1.46(1.26-1.63) | 0.68(0.35-1.02) |
|  | Southern Sub-Saharan Africa | 1,818.95(1,534.03-2,408.10) | 259.14(218.55-343.07) | 6,178.17(5,482.70-6,874.08) | 428.94(380.65-477.26) | 2.40(1.41-2.95) | 1.75(1.46-2.04) |
|  | Tropical Latin America | 4,644.98(4,209.25-4,937.24) | 191.78(173.79-203.85) | 21,460.82(18,047.40-23,335.51) | 252.53(212.37-274.59) | 3.62(3.26-3.87) | 1.20(1.07-1.33) |
|  | Western Europe | 35,580.43(31,619.02-37,551.70) | 142.15(126.33-150.03) | 118,248.31(95,199.36-131,735.88) | 271.75(218.78-302.75) | 2.32(2.00-2.55) | 2.55(2.41-2.70) |
|  | Western Sub-Saharan Africa | 8,531.19(7,450.20-9,788.48) | 405.57(354.18-465.34) | 21,573.99(18,654.44-25,303.13) | 486.88(420.99-571.04) | 1.53(1.09-1.89) | 0.61(0.56-0.65) |
| Mortality of CKD in the older adults Between 1,990 and 2,19 at the Global and Regional Level. EAPC, estimated annual percentage change; SDI, Sociodemographic Index; UI, uncertainty interval. EAPC is expressed as 95% UIs. | | | | | | | |
