# Supplementary material for: Global, Regional, and National Burden of chronic kidney disease in older adults from 1990 to 2021: Results from the Global Burden of Disease Study 2021
Source: PLoS One. 2026 Jul 31;21(7):e0354811. doi: 10.1371/journal.pone.0354811 (PMC13426924; doi:10.1371/journal.pone.0354811)
Supplement: S4 Table — (DOCX) [file pone.0354811.s004.docx]

| **S4 Table. GBD 2021 DALYs data tables (Global, 5SDI, 21 Regions)** | | | | | | | |
| --- | --- | --- | --- | --- | --- | --- | --- |
| Location | | Rate per 100 000 (95% UI) | |  |  |  |  |
|  |  | 1990 |  | 2021 |  | 1990-2021 | |
|  |  | DALYs cases | DALYs rate | DALYs cases | DALYs rate | Cases change | EAPCs |
| Global | | 3,226,423.86(2,893,419.57-3,591,165.21) | 2,749.50(2,465.72-3,060.33) | 10,621,485.62(9,430,923.12-11,652,072.63) | 3,681.25(3,268.62-4,038.44) | 2.29(1.96-2.50) | 1.07(1.02-1.12) |
| SDI | |  |  |  |  |  |  |
|  | High | 1,027,027.23(914,421.12-1,134,109.40) | 2,333.75(2,077.87-2,577.08) | 3,354,162.98(2,887,520.17-3,717,127.09) | 3,721.87(3,204.07-4,124.62) | 2.27(2.03-2.45) | 1.78(1.67-1.89) |
|  | High middle | 701,731.99(620,282.24-783,818.49) | 2,277.52(2,013.17-2,543.94) | 1,958,609.44(1,736,463.52-2,166,507.42) | 2,857.07(2,533.02-3,160.33) | 1.79(1.53-2.01) | 0.82(0.79-0.86) |
|  | Middle | 819,649.27(733,810.25-919,546.15) | 3,438.37(3,078.28-3,857.43) | 3,136,731.56(2,779,327.85-3,457,354.31) | 3,952.25(3,501.92-4,356.23) | 2.83(2.23-3.16) | 0.61(0.53-0.70) |
|  | Low middle | 453,542.75(392,381.26-547,400.02) | 3,254.17(2,815.34-3,927.60) | 1,565,390.46(1,407,767.11-1,767,990.75) | 4,050.34(3,642.50-4,574.56) | 2.45(1.77-2.86) | 0.68(0.62-0.73) |
|  | Low | 220,789.61(194,829.27-261,441.86) | 4,795.09(4,231.28-5,677.97) | 596,914.83(533,872.92-673,391.00) | 5,162.09(4,616.90-5,823.45) | 1.70(1.36-2.00) | 0.22(0.14-0.30) |
| Regions | |  |  |  |  |  |  |
|  | Andean Latin America | 29,287.33(26,253.52-32,778.54) | 4,831.15(4,330.70-5,407.05) | 134,464.74(112,212.93-158,649.41) | 6,765.11(5,645.59-7,981.88) | 3.59(2.77-4.54) | 1.15(0.86-1.44) |
|  | Australasia | 19,053.05(17,064.42-20,961.44) | 2,164.84(1,938.89-2,381.67) | 63,458.04(53,865.36-70,546.78) | 2,806.68(2,382.41-3,120.21) | 2.33(2.08-2.56) | 1.37(1.13-1.61) |
|  | Caribbean | 26,270.80(24,125.64-29,180.34) | 3,023.93(2,777.01-3,358.83) | 82,153.58(72,143.95-91,389.25) | 4,171.61(3,663.34-4,640.58) | 2.13(1.79-2.46) | 1.39(1.26-1.52) |
|  | Central Asia | 22,853.34(17,448.62-28,769.86) | 1,566.65(1,196.14-1,972.23) | 54,901.04(46,929.65-63,445.45) | 2,833.07(2,421.72-3,273.99) | 1.40(1.05-1.82) | 1.90(1.77-2.04) |
|  | Central Europe | 105,266.68(95,810.95-115,493.86) | 2,057.55(1,872.72-2,257.45) | 214,458.81(191,412.13-238,490.96) | 2,412.41(2,153.16-2,682.74) | 1.04(0.92-1.15) | 0.65(0.53-0.77) |
|  | Central Latin America | 120,549.82(111,890.61-128,865.04) | 5,119.11(4,751.40-5,472.22) | 530,021.18(476,360.13-585,159.94) | 6,494.82(5,837.27-7,170.49) | 3.40(3.04-3.72) | 1.33(0.98-1.68) |
|  | Central Sub-Saharan Africa | 24,441.78(20,465.72-29,418.76) | 6,979.44(5,844.06-8,400.63) | 72,013.74(55,014.26-92,356.97) | 7,487.01(5,719.63-9,602.02) | 1.95(1.18-2.78) | 0.15(0.07-0.24) |
|  | East Asia | 508,615.10(444,442.60-591,483.88) | 2,615.90(2,285.85-3,042.10) | 1,742,333.39(1,477,315.32-2,001,215.78) | 2,539.67(2,153.38-2,917.03) | 2.43(1.81-2.98) | 0.14(0.02-0.25) |
|  | Eastern Europe | 82,889.70(63,258.66-105,654.96) | 845.03(644.90-1,077.11) | 168,654.26(141,424.43-201,589.01) | 1,386.17(1,162.37-1,656.86) | 1.03(0.83-1.28) | 1.49(1.20-1.78) |
|  | Eastern Sub-Saharan Africa | 94,029.05(81,095.76-113,894.48) | 6,237.84(5,379.85-7,555.70) | 247,321.30(216,839.24-280,989.31) | 6,746.30(5,914.82-7,664.67) | 1.63(1.15-2.02) | 0.09(0.01-0.16) |
|  | High-income Asia Pacific | 199,396.17(177,981.37-214,798.12) | 2,949.64(2,632.86-3,177.48) | 703,482.48(581,389.70-790,226.76) | 3,077.32(2,543.23-3,456.77) | 2.53(2.19-2.75) | 0.06(-0.02-0.15) |
|  | High-income North America | 320,513.95(280,808.12-359,456.99) | 2,233.12(1,956.48-2,504.45) | 1,265,174.71(1,085,363.26-1,388,379.22) | 4,875.77(4,182.80-5,350.57) | 2.95(2.68-3.23) | 2.94(2.76-3.11) |
|  | North Africa and Middle East | 218,546.54(178,884.74-333,628.30) | 5,758.28(4,713.27-8,790.46) | 803,842.28(699,485.78-895,082.00) | 7,147.42(6,219.53-7,958.68) | 2.68(1.37-3.55) | 0.97(0.78-1.16) |
|  | Oceania | 1,363.82(1,097.81-1,766.95) | 2,910.89(2,343.13-3,771.31) | 5,102.53(4,337.84-6,201.62) | 3,648.95(3,102.10-4,434.94) | 2.74(1.88-3.78) | 0.85(0.80-0.91) |
|  | South Asia | 313,310.93(267,952.53-364,073.91) | 2,547.03(2,178.29-2,959.70) | 1,223,035.93(1,056,069.80-1,396,970.80) | 3,062.31(2,644.25-3,497.82) | 2.90(2.29-3.45) | 0.46(0.37-0.55) |
|  | Southeast Asia | 230,765.15(200,889.38-267,916.83) | 3,914.50(3,407.71-4,544.71) | 843,393.02(740,203.74-952,545.48) | 5,112.17(4,486.69-5,773.79) | 2.65(2.01-3.12) | 0.80(0.76-0.83) |
|  | Southern Latin America | 77,665.53(72,492.32-82,535.96) | 5,034.11(4,698.80-5,349.81) | 173,722.87(152,448.41-186,699.91) | 5,095.58(4,471.56-5,476.22) | 1.24(1.07-1.39) | 0.33(0.05-0.62) |
|  | Southern Sub-Saharan Africa | 28,392.99(24,275.29-35,714.90) | 4,045.00(3,458.38-5,088.12) | 89,904.55(80,542.49-101,256.61) | 6,241.92(5,591.92-7,030.07) | 2.17(1.39-2.61) | 1.50(1.24-1.75) |
|  | Tropical Latin America | 79,141.31(71,544.99-86,474.69) | 3,267.63(2,953.99-3,570.41) | 328,341.66(287,349.08-360,291.87) | 3,863.65(3,381.28-4,239.61) | 3.15(2.90-3.35) | 0.76(0.65-0.86) |
|  | Western Europe | 592,630.25(523,076.39-658,964.44) | 2,367.71(2,089.83-2,632.74) | 1,557,160.40(1,328,525.58-1,731,721.18) | 3,578.59(3,053.16-3,979.76) | 1.63(1.40-1.81) | 1.67(1.57-1.78) |
|  | Western Sub-Saharan Africa | 131,440.56(115,503.98-150,570.54) | 6,248.66(5,491.04-7,158.10) | 318,545.11(280,564.68-366,675.32) | 7,188.86(6,331.73-8,275.05) | 1.42(1.06-1.73) | 0.46(0.42-0.50) |
| DALYs of CKD in the older adults Between 1,990 and 2,19 at the Global and Regional Level. EAPC, estimated annual percentage change; SDI, Sociodemographic Index; UI, uncertainty interval. EAPC is expressed as 95% UIs. | | | | | | | |
