# Supplementary material for: Global, Regional, and National Burden of chronic kidney disease in older adults from 1990 to 2021: Results from the Global Burden of Disease Study 2021
Source: PLoS One. 2026 Jul 31;21(7):e0354811. doi: 10.1371/journal.pone.0354811 (PMC13426924; doi:10.1371/journal.pone.0354811)
Supplement: Attachment — Submitted filename: Response to Reviewers.docx [file pone.0354811.s006.docx]

**Response letter**

Dear editors:

Thank you for your letter and for the reviewers’ comments concerning your manuscript entitled “Global, Regional, and National Burden of Chronic Kidney Disease in Older Adults From 1990 to 2021: Results From the Global Burden of Disease Study 2021”. Actually, as for the suggestions of the Reviewers, the revised manuscript I have submitted. Those comments are all valuable and very helpful for revising and improving our paper, as well as the important guiding significance to our researches. We have studied comments carefully and have made correction which we hope meet with approval.

This letter is our point-by-point response to the comments raised by you and the reviewers. The comments are reproduced and our responses are given directly afterward using red text. We would like to thank you for allowing us to resubmit a revised copy of the manuscript. We hope the revised manuscript will meet the criteria and can be accepted for publication in PLOS ONE.

Thank you.

Table 1 Responses to the reviewers’ suggestions

| Reviewer | Suggestions | Revisions |
| --- | --- | --- |
| Editorial Office | 1. Please ensure that your manuscript meets PLOS ONE's style requirements, including those for file naming. | Special thanks to you for your useful suggestions. We have revised the manuscript formatting according to the “PLOS ONE MANUSCRIPT BODY FORMATTING GUIDELINES” and modified the file naming convention. |
|  | 2. Please include a separate caption for each figure in your manuscript. | Special thanks to you for your useful suggestions. We have revised the figure in the manuscript to improve readability. You can see our modifications in the newly uploaded figure. |
|  | 3. We note that Figure 4 in your submission contain [map/satellite] images which may be copyrighted. All PLOS content is published under the Creative Commons Attribution License (CC BY 4.0), which means that the manuscript, images, and Supporting Information files will be freely available online, and any third party is permitted to access, download, copy, distribute, and use these materials in any way, even commercially, with proper attribution. For these reasons, we cannot publish previously copyrighted maps or satellite images created using proprietary data, such as Google software (Google Maps, Street View, and Earth). | Special thanks to you for your useful suggestions. Below is the author's response.  a) Source of Map Images:  The map images in article were created using the rnaturalearth R package, which provides access to Natural Earth map data. This data is open-source and available at https://github.com/ropensci/rnaturalearth.  b) Copyright Status:  To our knowledge, the map data from Natural Earth provided through the rnaturalearth package is in the public domain and therefore not subject to copyright restrictions. Natural Earth map data is widely used for world mapping and is explicitly designed for open, unrestricted use.  c) Permission for Use:  Since the map images derive from open-source public domain data provided by Natural Earth, we believe no additional copyright permission is required. However, we can include a statement attributing Natural Earth as the data source if needed. |
|  | 4. If the reviewer comments include a recommendation to cite specific previously published works, please review and evaluate these publications to determine whether they are relevant and should be cited. There is no requirement to cite these works unless the editor has indicated otherwise. | Special thanks to you for your useful suggestions. We will only incorporate reference suggestions from other reviewers if we determine that they are highly relevant to my literature. |
| #1 | I read with interest the manuscript entitled “Global, Regional, and National Burden of Chronic 1 Kidney Disease in Older Adults From 1990 to 2021: Results From the Global Burden of Disease Study 2021”. The manuscript is well written and addresses a topic of interest. The study is based on data extracted from the Global Burden of Disease study 2021. The analysis included data from 204 countries that can provide data for CKD assessment, focusing on CKD in older adults aged 75 years and older between 2019 and 2021. The main results indicate a subtle decline in prevalence, accompanied by an increase in incidence, morbidity, and mortality during the study period. There are some publications based on changes in the burden of CKD in similar periods (listed below). However, there is only one preprint that focuses on this specific group of older adults, using a definition of older adults as those over 65 years (see below).  (1) Deng, L., Guo, S., Liu, Y. et al. Global, regional, and national burden of chronic kidney disease and its underlying etiologies from 1990 to 2021: a systematic analysis for the Global Burden of Disease Study 2021. BMC Public Health 25, 636 (2025). https://doi.org/10.1186/s12889-025-21851-z and  (2) Guo, J., Jiao, W., Xia, S. et al. The global, regional, and national patterns of change in the burden of chronic kidney disease from 1990 to 2021. BMC Nephrol 26, 136 (2025). https://doi.org/10.1186/s12882-025-04028-z  (3) Wang L, He Y, Han C, et al. Global burden of chronic kidney disease and risk factors, 1990–2021: an update from the global burden of disease study 2021. Front Public Health 2025; 13: 1542329.  (4) Xie K, Cao H, Ling S, et al. Global, regional, and national burden of chronic kidney disease, 1990-2021: a systematic analysis for the global burden of disease study 2021. Front Endocrinol (Lausanne) 2025; 16: 1526482.  (5) Preprint The Lancet collaboration with SSRN entitled Global Burden of Chronic Kidney Disease in Adults Aged 65 Years and Older, 1990-2021: A Systematic Analysis from the Global Burden of Disease Study 2021 from Xun-Liang Li et al. posted on Nov-2024 | Special thanks to you for your affirmation and useful suggestions. |
|  | My primary concern is that the results are merely descriptive, which limits our ability to draw more useful conclusions. Changes in CKD prevalence and incidence, DALYs, and mortality, as well as those discriminated against by region and SDI, have already been reported. The analysis is precise and well-executed, but some areas could benefit from further elaboration. Specifically, providing more details on the potential causes of the EAPCs' findings. The whole analysis results support the conclusions. However, | Special thanks to you for your useful suggestions. In response to your concerns, we have expanded the discussion section of the manuscript to include an analysis of the potential causes underlying our findings, thereby strengthening the analytical basis for our conclusions. You can see our revisions in the discussion and conclusion section of the manuscript. |
|  | In the Results section of the Summary, the following sentence states that prevalence initially decreases and then increases. Please clarify. “From 1990 to 2021, the prevalence of CKD in the older adults shows a decreasing trend, with a prevalence EAPCs of -0.06 (95%UI, -0.09--0.03); the prevalence 31 of CKD in the older adults shows an increasing trend, with EAPCs of 0.50 (95%UI, 0.47-0.53).” | Special thanks to you for your useful suggestions. We have revised the results section of the abstract. You can see our modifications on lines 32-34. The changes are as follows: “From 1990 to 2021, the prevalence of CKD in the older adults shows a decreasing trend, with EAPCs of -0.06 (95%UI, -0.09--0.03); the incidence of CKD in the older adults shows an increasing trend, with EAPCs of 0.50 (95%UI, 0.47-0.53).” |
|  | The manuscript shows results of big numbers (millions of people), in my opinion, and for better visualization of the readers, two decimals are not necessary, and a thousand separator is needed. | Special thanks to you for your useful suggestions. We have removed the decimal portions from larger numbers in the manuscript and added thousand separators to enhance readability for our readers. You can see our modifications in the manuscript. |
|  | End of Line 54 and start of Line 55 have a repeated phrase “older adults”, please correct. | Special thanks to you for your useful suggestions. We have removed the redundant older adults from the manuscript. |
|  | Figures: legends in figures are not readable. | Special thanks to you for your useful suggestions. We created new legends for the figures in the manuscript to ensure their readability. |
| #2 | However, I identified several weaknesses and limitations in your study:  1. The research relies on the GBD database, which contains data from individual countries. The accuracy and availability of the data may be affected by the quality of national registry data.  2. The study acknowledges the unknown number of undiagnosed cases of CKD in older adults, which may limit the analysis of the true burden of the disease.  3. There is a lack of detailed information on risk factors for CKD in older adults, which restricts the ability to analyze contributing factors comprehensively.  4. The GBD database categorizes CKD into five broad categories, including "chronic kidney disease due to other and unspecified causes." This classification may lead to wasted data and limit the study of specific etiologies and mechanisms of CKD. | Special thanks to you for your affirmation and useful suggestions. |
|  | Further, the study does not explicitly mention biases, but based on the limitations described, there some potential biases the authors should be aware of;  1. The study relies on data from the GBD database, which may not fully capture populations in countries with weaker data systems. This could lead to a selection bias and underrepresentation of certain regions or demographics.  2. Variability in the quality and completeness of national registry data may result in underreporting or misreporting of CKD cases.  3. The broad categorization of CKD causes (e.g., "other and unspecified causes") may obscure specific etiologies, leading to less precise analysis.  4. The study focuses on older adults aged 75 years and above, potentially overlooking trends and risk factors in younger age groups that could influence CKD progression in later years.  Addressing these biases in future research would require more granular data collection, improved classification systems, and efforts to include underrepresented populations. | Special thanks to you for your useful suggestions. We have revised the statement regarding limitations in the manuscript. The content is as follows:  This study has several limitations. First, our research and analysis are based on the GBD database, which incorporates data from various countries. Consequently, the accuracy and availability of the data may be influenced by the quality of national registry data, potentially leading to selection bias and underrepresentation of certain demographic groups in some regions. Second, variations in the quality and completeness of national registry data may result in underreporting and missed cases of CKD among older adults. Third, the unknown number of undiagnosed CKD cases in older adults and the lack of information on CKD risk factors in this population may obscure the disease's etiology, limiting our analysis of the disease burden. Fourth, the GBD database categorizes CKD into five groups, one titled “Chronic kidney disease due to other and unspecified causes.” Analysis revealed that this broad classification may result in data underutilization and hinder research into CKD etiology and mechanisms. Fifth, to better investigate the impact of age on elderly CKD, this study selected participants aged 75 years and older as subjects. This approach may have overlooked trends and risk factors potentially influencing the progression of elderly CKD in younger age groups. |

We have tried our best to improve the overall quality of the manuscript and have made some major changes in the revised manuscript. These changes have been marked in red in the revised paper.

We appreciate the Editors/Reviewers’ for their useful comments and sincerely hope that the corrections will be met with a positive response.

Once again, thank you very much for your great suggestions and comments.

Yours sincerely,

All authors
