# Supplementary material for: Global, Regional, and National Burden of chronic kidney disease in older adults from 1990 to 2021: Results from the Global Burden of Disease Study 2021
Source: PLoS One. 2026 Jul 31;21(7):e0354811. doi: 10.1371/journal.pone.0354811 (PMC13426924; doi:10.1371/journal.pone.0354811)
Supplement: Attachment — Submitted filename: Response_to_Reviewers_auresp_2.docx [file pone.0354811.s007.docx]

**Response letter**

Dear editors:

Thank you for your letter and for the reviewers’ comments concerning your manuscript entitled “Global, Regional, and National Burden of Chronic Kidney Disease in Older Adults From 1990 to 2021: Results From the Global Burden of Disease Study 2021”. We sincerely thank the reviewers for their valuable comments, which have greatly improved our manuscript. We have carefully revised the paper to address all points raised and respectfully request approval of the updated manuscript.

This letter is our point-by-point response to the comments raised by you and the reviewers. The comments are reproduced and our responses are given directly afterward using red text. We would like to thank you for allowing us to resubmit a revised copy of the manuscript. We hope the revised manuscript will meet the criteria and can be accepted for publication in PLOS ONE.

Thank you.

Table 1 Responses to the reviewers’ suggestions

| Reviewer | Suggestions | Revisions |
| --- | --- | --- |
| Editorial Office | 1. If the reviewer comments include a recommendation to cite specific previously published works, please review and evaluate these publications to determine whether they are relevant and should be cited. There is no requirement to cite these works unless the editor has indicated otherwise. | Special thanks to you for your useful suggestions. My co-authors and I shall rigorously examine all relevant literature, considering citations only where they demonstrably enhance the manuscript's readability. |
|  | 2. Please review your reference list to ensure that it is complete and correct. If you have cited papers that have been retracted, please include the rationale for doing so in the manuscript text, or remove these references and replace them with relevant current references. Any changes to the reference list should be mentioned in the rebuttal letter that accompanies your revised manuscript. If you need to cite a retracted article, indicate the article’s retracted status in the References list and also include a citation and full reference for the retraction notice. | Special thanks to you for your useful suggestions. We have re-examined all references to ensure their accuracy. Concurrently, we have revised the references within the manuscript to ensure compliance with PLOS formatting requirements. |
| #2 | The authors made several specific changes in response to the reviewers' comments, as outlined in their response letter. Here are the key changes:  Formatting and Style Adjustments  1. Manuscript Formatting: Revised to meet PLOS ONE's style requirements, including file naming conventions and formatting guidelines.  2. Figure Captions: Separate captions were added for each figure to improve clarity.  3. Figure Legends: Updated to ensure readability, addressing concerns about the previous legends being unclear.  4. Number Formatting: Large numbers were formatted with thousand separators, and unnecessary decimals were removed for better visualization.  Clarifications in Results  1. Prevalence and Incidence Trends: The authors clarified the sentence in the results section of the abstract to avoid confusion. The revised text now accurately states:  "From 1990 to 2021, the prevalence of CKD in the older adults shows a decreasing trend, with EAPCs of -0.06 (95%UI, -0.09--0.03); the incidence of CKD in the older adults shows an increasing trend, with EAPCs of 0.50 (95%UI, 0.47-0.53)."  2. Redundant Phrases: Removed repeated phrases like "older adults" in the introduction section.  Expanded Discussion  1. Potential Causes of Findings: The discussion section was expanded to analyze the potential causes underlying the Estimated Annual Percentage Changes (EAPCs) findings, strengthening the analytical basis for conclusions.  2. Biases and Limitations: The limitations section was revised to explicitly address biases related to data accuracy, underreporting, and broad categorization in the GBD database. The authors acknowledged the challenges of relying on national registry data and the potential underrepresentation of certain demographics.  Response to Reviewer Suggestions  1. Clarification of Trends: The authors clarified trends in CKD prevalence and incidence in the abstract and results sections.  2. Improved Number Presentation: Removed decimals from large numbers and added thousand separators for better visualization.  3. Addressing Biases: Expanded the limitations section to include potential biases and challenges in data collection and analysis.  Acknowledgment of Reviewer Suggestions  1. Incorporation of Relevant References: The authors evaluated suggested references and incorporated them if deemed relevant.  2. Future Research Directions: Suggestions for addressing biases in future research were included, such as improved classification systems and efforts to include underrepresented populations.  These changes demonstrate that the authors carefully considered and addressed all reviewer comments, improving the manuscript's clarity, readability, and analytical depth. | Special thanks to you for your affirmation and useful suggestions. |
| #3 | The authors have made a clear effort to address the points raised in the previous round, and the manuscript is notably improved. The issues around formatting, clarity, and internal consistency have largely been resolved, and the paper is now much easier to read and interpret. In particular, the abstract has been corrected so that the trends in prevalence and incidence are no longer contradictory, the large numbers are formatted in a more reader-friendly way, repeated wording has been removed, and the figure legends are now legible.  I also appreciate the expanded Discussion and the more explicit limitations section. The authors now acknowledge the dependence on registry quality, the likelihood of underdiagnosis and underreporting, the lack of risk factor detail, the broad etiological categories used in the database, and the fact that focusing on people aged 75 and over may miss earlier determinants of disease. This transparency strengthens the paper and addresses most of the conceptual concerns raised previously. | Special thanks to you for your affirmation and useful suggestions. |
|  | That said, there are still a few points that I think would benefit from further clarification before publication.  1.The manuscript would benefit from a clearer statement of what is new here relative to existing global CKD burden studies. The focus on adults aged 75 years and older is important, but this is not highlighted strongly enough as the key contribution. I would encourage the authors to add one or two sentences in the Introduction or early Discussion explaining explicitly how this analysis differs from, and adds to, prior GBD-based work. | Special thanks to you for your useful suggestions. We have revised the introduction section of the manuscript. Our amendments can be found on lines 71–73 and 83–89 of the submitted manuscript, as follows:  Lines 71-73：Moreover, the greater the age of CKD in the older adults, the more significant the impact on themselves, their families, finances, and indeed society at large.  Lines 83-89：Finally, our study distinguishes itself from previous research on CKD across all age groups or single-etiology CKD by focusing specifically on elderly CKD patients aged 75 years and above. This enables us to understand the impact of age on the disease burden of CKD, while also partially filling gaps in research concerning the disease burden of CKD in the older-older adults. We also hope to advance progress in the prevention and treatment of CKD in the older adults, ultimately providing a theoretical basis for alleviating the burden of age-related health losses associated with CKD. |
|  | 2.The description of the study population and age groups could be made more consistent. The Methods mention different concepts of “older” adults, but the analysis appears to focus on those aged 75 and above. This should be stated very clearly, and any background references to other age groups should be either clarified or removed to avoid confusion. | Special thanks to you for your useful suggestions. We have revised the Overview and Data Collection section of the manuscript. Our amendments can be found on lines 97–101 of the submitted manuscript and are as follows: With advances in public health technology and increased life expectancy, the age of 65 has become insufficient for distinguishing the elderly population. Individuals aged 65 to 74 are now termed young older adults, whose physical and mental attributes align more closely with middle-aged adults than with the actual older adults. |
|  | 3.The statistical methods are described only briefly. While this is not a methods paper, readers would benefit from a little more detail on how EAPCs were estimated, whether crude or age-standardized rates were used for each outcome, and how uncertainty was handled. A short expansion of this section would improve reproducibility. | Special thanks to you for your useful suggestions. We have added a section entitled “GBD Data Estimation Methodology” to the Methods section of our manuscript. This can be found on lines 133–147 of our submitted manuscript, with the new content as follows:  GBD Data Estimation Methodology  The GBD database draws on a comprehensive repository of over 100 thousand data sources worldwide—including vital registration systems, population censuses, household surveys, and healthcare records—to estimate the prevalence, incidence, mortality, and DALYs for diseases and injuries. The DALYs are calculated by summing the Years Lived with Disability (YLDs) and Years of Life Lost (YLLs). Specifically, YLDs are derived by multiplying the cause-, age-, sex-, location-, and year-specific prevalence of disease sequelae by their corresponding disability weights. YLLs are computed by multiplying the number of cause-specific deaths in each demographic stratum by the standard life expectancy at the age of deaths[37]. Estimated Annual Percentage Changes (EAPCs) represent the average annual trend in Age-Standardized Rate (ASR) over a specific time period. An estimated value and lower bound of the 95% UI > 0 indicate an upward trend in the ASR; an estimated value and upper bound of the 95% UI < 0 indicate a downward trend. If neither condition is met, the trend is considered stable. |
|  | 4.some of the interpretations of correlations (for example between SDI and DALYs or mortality) are plausible but somewhat strong given the ecological nature of the data. It would be helpful to add a short caution that these associations are not causal and may reflect confounding by healthcare access, diagnostic practices, survival, and reporting differences.  Related to this, there are places where levels and trends are discussed together in a way that could confuse readers for example, a region may have a high current burden but a slower growth rate, or vice versa. Making this distinction explicit in the text would improve clarity. | Special thanks to you for your useful suggestions. We have revised the discussion section of the manuscript and incorporated non-causality prompts. |
|  | Finally, although the authors have explained the source and licensing of the map data in their response, it would be safer to include a brief attribution directly in the figure captions so that this information is visible in the published article. | Special thanks to you for your useful suggestions. We have updated the uploaded Figure 4 and added a source attribution beneath the figure to ensure clearer presentation within the manuscript. |
|  | A final light language edit would also help smooth some phrasing, but this is a minor point. | Special thanks to you for your useful suggestions. We have endeavoured to refine the phrasing to enhance the readability of the article. |

We have tried our best to improve the overall quality of the manuscript and have made some major changes in the revised manuscript. These changes have been marked in red in the revised paper.

We appreciate the Editors/Reviewers’ for their useful comments and sincerely hope that the corrections will be met with a positive response.

Once again, thank you very much for your great suggestions and comments.

Yours sincerely,

All authors
