# Supplementary material for: Global, Regional, and National Burden of chronic kidney disease in older adults from 1990 to 2021: Results from the Global Burden of Disease Study 2021
Source: PLoS One. 2026 Jul 31;21(7):e0354811. doi: 10.1371/journal.pone.0354811 (PMC13426924; doi:10.1371/journal.pone.0354811)
Supplement: Attachment — Submitted filename: Response_to_Reviewers_auresp_3.docx [file pone.0354811.s008.docx]

**Response letter**

Dear editors:

Thank you for your letter and for the reviewers’ comments concerning your manuscript entitled “Global, Regional, and National Burden of Chronic Kidney Disease in Older Adults From 1990 to 2021: Results From the Global Burden of Disease Study 2021”. We sincerely thank the reviewers for their valuable comments, which have greatly improved our manuscript. We have carefully revised the paper to address all points raised and respectfully request approval of the updated manuscript.

This letter is our point-by-point response to the comments raised by you and the reviewers. The comments are reproduced and our responses are given directly afterward using red text. We would like to thank you for allowing us to resubmit a revised copy of the manuscript. We hope the revised manuscript will meet the criteria and can be accepted for publication in PLOS ONE.

Thank you.

Table 1 Responses to the reviewers’ suggestions

| Reviewer | Suggestions | Revisions |
| --- | --- | --- |
| #3 | 1. The study remains primarily descriptive and is essentially a subgroup analysis of publicly available GBD data limited to individuals aged 75 years and older. While this age-focused perspective is relevant, the manuscript does not convincingly demonstrate how this approach substantially advances existing knowledge from prior global CKD burden studies.  The analysis does not introduce new modeling strategies, decomposition approaches, or age–period–cohort analysis. Nor does it provide deeper mechanistic or policy-level insight beyond descriptive trends. The authors should strengthen the justification for the study’s added value and clarify what new understanding emerges specifically from focusing on this age group. | Special thanks to your useful suggestions. We are very grateful to the reviewers for raising this key concern. We fully agree that it is essential to clarify the unique value of this study. In our revisions, we have strengthened this argument in several key sections, explicitly elevating the ‘subgroup analysis’ to a scientific exploration aimed at revealing ‘unique epidemiological patterns’. Specific revisions include: stating at the outset that this study is the ‘first systematic assessment’ and clarifying that its purpose is to ‘reveal epidemiological patterns unique to this population’; and emphasising in the concluding summary that the study provides evidence for ‘targeted interventions’ and ‘resource allocation’. In the Discussion section, we state unequivocally that this study reveals “unique epidemiological patterns distinct from those observed in the general population or younger elderly cohorts”, and identify the “trend divergence phenomenon” as a core new finding. We then provide an in-depth analysis of two key contradictions: the “paradox of CKD in the very elderly” (stable prevalence versus soaring mortality) and the “paradox of high SDI regions” (the fastest-rising mortality rates in high SDI areas), exploring their underlying physiological, social and healthcare system drivers. This goes beyond a simple description of trends to offer a mechanistic interpretation of the shifting dynamics of the disease. Finally, in the conclusion, we explicitly propose differentiated strategies: high-SDI regions must shift towards curbing the rise in mortality, whilst low-SDI regions must strengthen early diagnosis and management. This provides clear insights at the policy level. |
|  | 2. Although a section describing GBD methodology has been added, several important methodological points remain unclear:  Whether all reported rates are age-standardized, or if crude and standardized rates are mixed.  The exact regression specification used for EAPC estimation (e.g., log-linear modeling).  Whether assumptions underlying linear trend estimation were assessed.  How statistical uncertainty was incorporated into the analyses.  For transparency and reproducibility, these details should be explicitly described. | Special thanks to you for your useful suggestions. We thank the reviewers for highlighting the importance of methodological details. To ensure full transparency and reproducibility, we have added the following detailed explanation to the ‘Statistical Analysis’ subsection (lines 149-191):  **Standardization of rates:** We have explicitly stated that ‘all rates reported in this study are age-standardized’ and explained the rationale for standardizing even within the ≥75 age group (lines 151-158).  **EAPC regression model:** We have provided the specific logarithmic linear regression formula ln(ASR) = α + β × Calendar Year + ε, as well as the formula for calculating the EAPC: EAPC = (e^β – 1) × 100% (lines 159-166).  **Assumption of a linear trend:** We explain the rationale for uniformly adopting a log-linear model to maintain cross-national comparability, whilst acknowledging that this is a methodological simplification which may fail to capture non-linear trends; this is further elaborated in the ‘Limitations’ section (lines 167-175 and 360-366).  **Statistical uncertainty:** We clarify that all 95% uncertainty intervals (UI) are derived directly from the official GBD modelling results, and provide a detailed explanation of how the EAPC UI is calculated based on the rate UI, as well as the specific criteria for trend determination (lines 176-187). |
|  | 3. There are still numerical and typographical inconsistencies, including irregular formatting of large numbers and misplaced digits in some sections (including the abstract). These errors are concerning at the R2 stage and raise questions about data verification.  The authors should carefully recheck: Case numbers, Rates, Confidence intervals, EAPC values. A comprehensive numerical audit is necessary. | Special thanks to you for your useful suggestions. We sincerely apologize for the inconsistencies in data formatting in previous versions and have now rectified this issue thoroughly. As part of this revision, we have conducted a comprehensive numerical audit to ensure that: all numbers greater than 999 throughout the text have been formatted with a thousands separator (e.g. 126,963,771); and that all case numbers, rates, 95% UIs and EAPC values in the abstract, main text, figures and tables, and the ‘Results’ section are fully consistent and expressed in accordance with standard conventions (95% UI: lower limit–upper limit). |
|  | 4. The Results section frequently enumerates country-level findings without sufficient analytical synthesis. This reduces the interpretive value of the manuscript and makes the section difficult to follow.  The authors should reduce repetitive listing of country statistics and instead emphasize broader patterns, regional contrasts, and key comparative insights. The current presentation reads more like a data summary than an analytical epidemiological study. | Special thanks to you for your useful suggestions. We fully agree with the reviewers’ comments and have restructured the ‘Results’ section, transforming it from a ‘list of data’ into a ‘report of patterns’.  Firstly, we have chosen to consolidate the original statements on regions and countries. Secondly, in the section on geographical regions (lines 257-277), we have significantly reduced the itemised listing of the 21 regions. Instead, we summarized the extreme distribution of the burden and compared differences in rates of change (for example, Latin America in the Andes region had the fastest-growing incidence, whilst sub-Saharan Africa in East Africa had the slowest-growing mortality rate). At the same time, we emphasized the overall pattern that the burden in most regions is higher than the global average.  In the section on countries (lines 280-289): We no longer list specific country names (except for China as an example of the highest number of cases, and a few typical countries with the highest burden rates), but instead use statistical descriptions such as “the mortality rate in the vast majority of countries (169/204, 82.8%) is higher than the global average” to highlight the widespread nature and inequality of the disease burden.  These revisions make the results section focused and easy to understand, and provide a clear basis for subsequent discussion. |
|  | 5. Although the discussion has been moderated, some passages still suggest explanatory mechanisms (particularly regarding SDI and aging) that go beyond what can be supported by ecological, descriptive data. Interpretations should remain clearly framed within the study design. | Special thanks to you for your useful suggestions. We have reviewed and refined the wording in the discussion section to ensure that all explanations are couched in speculative and cautious terms.  For example, when analysing the ‘paradox of high SDI areas’ and the correlation between SDI and disease burden (lines 322-333), we have used phrases such as ‘may be related to’, ‘could reflect’ and ‘likely stems from’, and have listed several possible competing explanations (such as deep ageing, diagnostic differences and data quality).  Furthermore, we have clarified the limitations of the study design in the ‘Limitations’ section to prevent readers from drawing erroneous causal inferences. |
|  | 6. The manuscript would benefit from further language polishing. There are still grammatical inconsistencies and redundant phrasing that affect clarity. | Special thanks to you for your useful suggestions. We have carefully edited the entire manuscript, including, but not limited to, checking and correcting issues such as tense, subject-verb agreement and the use of articles; removing redundant and repetitive phrasing to make the sentences more concise and impactful; and optimising the transitions between paragraphs and sentences to enhance the logical flow of the manuscript. |

We have tried our best to improve the overall quality of the manuscript and have made some major changes in the revised manuscript. These changes have been marked in red in the revised paper.

We appreciate the Editors/Reviewers’ for their useful comments and sincerely hope that the corrections will be met with a positive response.

Once again, thank you very much for your great suggestions and comments.

Yours sincerely,

All authors
