# Supplementary material for: Global, Regional, and National Burden of chronic kidney disease in older adults from 1990 to 2021: Results from the Global Burden of Disease Study 2021
Source: PLoS One. 2026 Jul 31;21(7):e0354811. doi: 10.1371/journal.pone.0354811 (PMC13426924; doi:10.1371/journal.pone.0354811)
Supplement: Attachment — Submitted filename: Response_to_Reviewers_auresp_4.docx [file pone.0354811.s009.docx]

**Response letter**

Dear editors:

Thank you for your letter and for the reviewers’ comments concerning your manuscript entitled “Global, Regional, and National Burden of Chronic Kidney Disease in Older Adults From 1990 to 2021: Results From the Global Burden of Disease Study 2021”. We sincerely thank the reviewers for their valuable comments, which have greatly improved our manuscript. We have carefully revised the paper to address all points raised and respectfully request approval of the updated manuscript.

This letter is our point-by-point response to the comments raised by you and the reviewers. The comments are reproduced and our responses are given directly afterward using red text. We would like to thank you for allowing us to resubmit a revised copy of the manuscript. We hope the revised manuscript will meet the criteria and can be accepted for publication in PLOS ONE.

Thank you.

Table 1 Responses to the reviewers’ suggestions

| Reviewer | Suggestions | Revisions |
| --- | --- | --- |
| #3 | 1. The manuscript has improved significantly, especially in the methods and discussion sections, and most of the major reviewer concerns have been addressed well. The added explanations on data sources, statistical methods, and study limitations have strengthened the paper considerably. | We sincerely thank the reviewer for this positive assessment. As suggested, we have maintained and further clarified the methodological rigor throughout the manuscript. |
|  | 2. The results section is clearer than before, but it still feels too descriptive in some parts, with many regional and country-specific examples. Focusing more on the key global patterns and reducing repetitive details would improve readability and overall impact. | Special thanks to you for your useful suggestions. We have substantially rewritten the “Geographic Regional and National-Level Trends” subsection in the Results to move from a listing of examples to a synthesis of overarching patterns.  For regional trends (lines 252-268), we now open with a synthesized statement: “The burden of CKD among older adults exhibited profound geographic disparities. In 2021, the highest age-standardized rates were concentrated in different regions...”We then summarize the most salient contrasts in temporal trends, stating: “Temporal trends (1990–2021) also varied markedly. While the age-standardized incidence of CKD increased in all 21 GBD regions, the growth was most rapid in Andean Latin America... and slowest in High-income North America...”This approach highlights the most illustrative comparisons rather than enumerating all regions.  For national-level findings (lines 271-279), we have eliminated the list of country names for each metric. Instead, we present a powerful summary statistic: “Critically, the distribution of the mortality burden was highly skewed. In 2021, the vast majority of countries (169 out of 204, 82.8%) had age-standardized mortality rates above the global average..., underscoring the pervasiveness of elevated CKD-related death risk...”This change shifts the focus from specific examples to the global scale of inequality, which is the key message. |
|  | 3. The discussion is much stronger now, particularly in explaining important findings such as the prevalence–mortality divergence and SDI-related differences. However, some sections could still be shortened and simplified to make the main messages clearer. | Special thanks to you for your useful suggestions. We have streamlined the Discussion to sharpen its focus on the two core paradoxical findings and their interpretation.  We have tightened the opening paragraph (lines 284-291) to immediately establish the study’s context and its central “high-age CKD paradox”.  We have consolidated the explanation of the “high-SDI region paradox” (lines 292-325). Previously, the discussion of SDI correlations, mortality trends, and the role of diabetes (like comorbidities) was more segmented. We have now integrated these points into a more fluid and logical argument. The revised text directly links the fastest mortality growth in high-SDI regions to advanced aging, contrasts it with the positive incidence-SDI correlation (possibly reflecting detection intensity), and incorporates diabetes as a key exemplar of the prevalent risk factors in these settings. This creates a more cohesive and simplified narrative around this paradox.  Throughout, we have pruned redundant phrases and simplified sentence structures to enhance readability and ensure the primary insights about divergent trends and SDI-related disparities stand out with maximum clarity. |
|  | 4. While the language has improved, the manuscript would still benefit from careful final editing to correct awkward phrasing, reduce repetition, and improve sentence flow. This would make the paper easier to read. | Special thanks to you for your useful suggestions. we have undertaken a meticulous, line-by-line edit of the entire manuscript. This process included:  Correcting awkward phrasing for more natural and precise academic English.  Reducing repetition of words and concepts, particularly in the Results and Discussion sections.  Improving sentence flow by varying sentence structure, ensuring clear pronoun references, and using transitional phrases to guide the reader logically from one idea to the next.  Verifying terminology consistency (e.g., standardized use of “older adults,” “age-standardized rate,” “Sociodemographic Index (SDI)”).  Correcting minor grammatical errors and ensuring proper punctuation. For example, the incorrect use of ‘-’ between numbers has been corrected and replaced with ‘–’, which complies with the formatting standards.  We are confident that this comprehensive language edit has significantly enhanced the fluency, professionalism, and overall readability of the manuscript. |

We have tried our best to improve the overall quality of the manuscript and have made some major changes in the revised manuscript. These changes have been marked in red in the revised paper.

We appreciate the Editors/Reviewers’ for their useful comments and sincerely hope that the corrections will be met with a positive response.

Once again, thank you very much for your great suggestions and comments.

Yours sincerely,

All authors
