# Supplementary material for: Global, Regional, and National Burden of chronic kidney disease in older adults from 1990 to 2021: Results from the Global Burden of Disease Study 2021
Source: PLoS One. 2026 Jul 31;21(7):e0354811. doi: 10.1371/journal.pone.0354811 (PMC13426924; doi:10.1371/journal.pone.0354811)
Supplement: Peer review history

# Peer review: Decision Letter 0

**DOI:** 10.1371/journal.pone.0354811.r001

**Authors:** Pasyodun Koralage Buddhika Mahesh

24 Sep 2025

PONE-D-25-28504

Global, Regional, and National Burden of Chronic Kidney Disease in Older Adults From 1990 to 2021: Results From the Global Burden of Disease Study 2021

PLOS ONE

Dear Dr. Shang,

Thank you for submitting your manuscript to PLOS ONE. After careful consideration, we feel that it has merit but does not fully meet PLOS ONE’s publication criteria as it currently stands. Therefore, we invite you to submit a revised version of the manuscript that addresses the points raised during the review process.

Please submit your revised manuscript by Nov 08 2025 11:59PM. If you will need more time than this to complete your revisions, please reply to this message or contact the journal office at plosone@plos.org. When you're ready to submit your revision, log on to https://www.editorialmanager.com/pone/ and select the 'Submissions Needing Revision' folder to locate your manuscript file.

Please include the following items when submitting your revised manuscript:

- A rebuttal letter that responds to each point raised by the academic editor and reviewer(s). You should upload this letter as a separate file labeled 'Response to Reviewers'.
- A marked-up copy of your manuscript that highlights changes made to the original version. You should upload this as a separate file labeled 'Revised Manuscript with Track Changes'.
- An unmarked version of your revised paper without tracked changes. You should upload this as a separate file labeled 'Manuscript'.

If you would like to make changes to your financial disclosure, please include your updated statement in your cover letter. Guidelines for resubmitting your figure files are available below the reviewer comments at the end of this letter.

If applicable, we recommend that you deposit your laboratory protocols in protocols.io to enhance the reproducibility of your results. Protocols.io assigns your protocol its own identifier (DOI) so that it can be cited independently in the future. For instructions see: https://journals.plos.org/plosone/s/submission-guidelines#loc-laboratory-protocols. Additionally, PLOS ONE offers an option for publishing peer-reviewed Lab Protocol articles, which describe protocols hosted on protocols.io. Read more information on sharing protocols at https://plos.org/protocols?utm_medium=editorial-email&utm_source=authorletters&utm_campaign=protocols.

We look forward to receiving your revised manuscript.

Kind regards,

Pasyodun Koralage Buddhika Mahesh

Academic Editor

PLOS ONE

Journal requirements:

When submitting your revision, we need you to address these additional requirements.

1. Please ensure that your manuscript meets PLOS ONE's style requirements, including those for file naming. The PLOS ONE style templates can be found at

https://journals.plos.org/plosone/s/file?id=wjVg/PLOSOne_formatting_sample_main_body.pdf and

https://journals.plos.org/plosone/s/file?id=ba62/PLOSOne_formatting_sample_title_authors_affiliations.pdf

2. Please include a separate caption for each figure in your manuscript.

3. We note that Figure 4 in your submission contain [map/satellite] images which may be copyrighted. All PLOS content is published under the Creative Commons Attribution License (CC BY 4.0), which means that the manuscript, images, and Supporting Information files will be freely available online, and any third party is permitted to access, download, copy, distribute, and use these materials in any way, even commercially, with proper attribution. For these reasons, we cannot publish previously copyrighted maps or satellite images created using proprietary data, such as Google software (Google Maps, Street View, and Earth). For more information, see our copyright guidelines: http://journals.plos.org/plosone/s/licenses-and-copyright.

We require you to either (1) present written permission from the copyright holder to publish these figures specifically under the CC BY 4.0 license, or (2) remove the figures from your submission:

1. You may seek permission from the original copyright holder of Figure 4 to publish the content specifically under the CC BY 4.0 license.

We recommend that you contact the original copyright holder with the Content Permission Form (http://journals.plos.org/plosone/s/file?id=7c09/content-permission-form.pdf) and the following text:

“I request permission for the open-access journal PLOS ONE to publish XXX under the Creative Commons Attribution License (CCAL) CC BY 4.0 (http://creativecommons.org/licenses/by/4.0/). Please be aware that this license allows unrestricted use and distribution, even commercially, by third parties. Please reply and provide explicit written permission to publish XXX under a CC BY license and complete the attached form.”

Please upload the completed Content Permission Form or other proof of granted permissions as an "Other" file with your submission.

In the figure caption of the copyrighted figure, please include the following text: “Reprinted from [ref] under a CC BY license, with permission from [name of publisher], original copyright [original copyright year].”

2. If you are unable to obtain permission from the original copyright holder to publish these figures under the CC BY 4.0 license or if the copyright holder’s requirements are incompatible with the CC BY 4.0 license, please either i) remove the figure or ii) supply a replacement figure that complies with the CC BY 4.0 license. Please check copyright information on all replacement figures and update the figure caption with source information. If applicable, please specify in the figure caption text when a figure is similar but not identical to the original image and is therefore for illustrative purposes only.

The following resources for replacing copyrighted map figures may be helpful:

USGS National Map Viewer (public domain): http://viewer.nationalmap.gov/viewer/

The Gateway to Astronaut Photography of Earth (public domain): http://eol.jsc.nasa.gov/sseop/clickmap/

Maps at the CIA (public domain): https://www.cia.gov/library/publications/the-world-factbook/index.html and https://www.cia.gov/library/publications/cia-maps-publications/index.html

NASA Earth Observatory (public domain): http://earthobservatory.nasa.gov/

Landsat: http://landsat.visibleearth.nasa.gov/

USGS EROS (Earth Resources Observatory and Science (EROS) Center) (public domain): http://eros.usgs.gov/#

Natural Earth (public domain): http://www.naturalearthdata.com/

4. If the reviewer comments include a recommendation to cite specific previously published works, please review and evaluate these publications to determine whether they are relevant and should be cited. There is no requirement to cite these works unless the editor has indicated otherwise.

Additional Editor Comments:

Reviewer #1:

Reviewer #2:

[Note: HTML markup is below. Please do not edit.]

Reviewers' comments:

Reviewer's Responses to Questions

**Comments to the Author**

1. Is the manuscript technically sound, and do the data support the conclusions?

The manuscript must describe a technically sound piece of scientific research with data that supports the conclusions. Experiments must have been conducted rigorously, with appropriate controls, replication, and sample sizes. The conclusions must be drawn appropriately based on the data presented.

Reviewer #1: Yes

Reviewer #2: Yes

**********

2. Has the statistical analysis been performed appropriately and rigorously?

Reviewer #1: Yes

Reviewer #2: Yes

**********

3. Have the authors made all data underlying the findings in their manuscript fully available?

The PLOS Data policy requires authors to make all data underlying the findings described in their manuscript fully available without restriction, with rare exception (please refer to the Data Availability Statement in the manuscript PDF file). The data should be provided as part of the manuscript or its supporting information, or deposited to a public repository. For example, in addition to summary statistics, the data points behind means, medians and variance measures should be available. If there are restrictions on publicly sharing data—e.g. participant privacy or use of data from a third party—those must be specified.

Reviewer #1: Yes

Reviewer #2: Yes

**********

4. Is the manuscript presented in an intelligible fashion and written in standard English?

PLOS ONE does not copyedit accepted manuscripts, so the language in submitted articles must be clear, correct, and unambiguous. Any typographical or grammatical errors should be corrected at revision, so please note any specific errors here.

Reviewer #1: Yes

Reviewer #2: Yes

**********

5. Review Comments to the Author

Please use the space provided to explain your answers to the questions above. You may also include additional comments for the author, including concerns about dual publication, research ethics, or publication ethics. (Please upload your review as an attachment if it exceeds 20,000 characters)

Reviewer #1: I read with interest the manuscript entitled “Global, Regional, and National Burden of Chronic 1 Kidney Disease in Older Adults From 1990 to 2021: Results From the Global Burden of Disease Study 2021”. The manuscript is well written and addresses a topic of interest. The study is based on data extracted from the Global Burden of Disease study 2021. The analysis included data from 204 countries that can provide data for CKD assessment, focusing on CKD in older adults aged 75 years and older between 2019 and 2021. The main results indicate a subtle decline in prevalence, accompanied by an increase in incidence, morbidity, and mortality during the study period. There are some publications based on changes in the burden of CKD in similar periods (listed below). However, there is only one preprint that focuses on this specific group of older adults, using a definition of older adults as those over 65 years (see below).

(1) Deng, L., Guo, S., Liu, Y. et al. Global, regional, and national burden of chronic kidney disease and its underlying etiologies from 1990 to 2021: a systematic analysis for the Global Burden of Disease Study 2021. BMC Public Health 25, 636 (2025). https://doi.org/10.1186/s12889-025-21851-z and

(2) Guo, J., Jiao, W., Xia, S. et al. The global, regional, and national patterns of change in the burden of chronic kidney disease from 1990 to 2021. BMC Nephrol 26, 136 (2025). https://doi.org/10.1186/s12882-025-04028-z

(3) Wang L, He Y, Han C, et al. Global burden of chronic kidney disease and risk factors, 1990–2021: an update from the global burden of disease study 2021. Front Public Health 2025; 13: 1542329.

(4) Xie K, Cao H, Ling S, et al. Global, regional, and national burden of chronic kidney disease, 1990-2021: a systematic analysis for the global burden of disease study 2021. Front Endocrinol (Lausanne) 2025; 16: 1526482.

(5) Preprint The Lancet collaboration with SSRN entitled Global Burden of Chronic Kidney Disease in Adults Aged 65 Years and Older, 1990-2021: A Systematic Analysis from the Global Burden of Disease Study 2021 from Xun-Liang Li et al. posted on Nov-2024

Some other aspects of the manuscript deserve attention_

• My primary concern is that the results are merely descriptive, which limits our ability to draw more useful conclusions. Changes in CKD prevalence and incidence, DALYs, and mortality, as well as those discriminated against by region and SDI, have already been reported. The analysis is precise and well-executed, but some areas could benefit from further elaboration. Specifically, providing more details on the potential causes of the EAPCs' findings. The whole analysis results support the conclusions. However,

• In the Results section of the Summary, the following sentence states that prevalence initially decreases and then increases. Please clarify. “From 1990 to 2021, the prevalence of CKD in the older adults shows a decreasing trend, with a prevalence EAPCs of -0.06 (95%UI, -0.09--0.03); the prevalence 31 of CKD in the older adults shows an increasing trend, with EAPCs of 0.50 (95%UI, 0.47-0.53).”

• The manuscript shows results of big numbers (millions of people), in my opinion, and for better visualization of the readers, two decimals are not necessary, and a thousand separator is needed.

• End of Line 54 and start of Line 55 have a repeated phrase “older adults”, please correct.

• Figures: legends in figures are not readable.

Reviewer #2: First of all, I would like to congratulate the authors for doing this all important study on CKD using the GBD data from 2021.

This study analyzes the global, regional, and national burden of chronic kidney disease (CKD) in older adults aged 75+ from 1990 to 2021 using data from the Global Burden of Disease Study 2021.

The study highlights significant regional and demographic disparities, emphasizing the need for tailored interventions to address CKD in aging populations.

The methodology of this study appears robust and well-structured, leveraging data from the Global Burden of Disease (GBD) Study 2021, which is a comprehensive and widely recognized database. Overall, the methodology is strong but could benefit from addressing data gaps and refining classifications for deeper insights.

However, I identified several weaknesses and limitations in your study:

1. The research relies on the GBD database, which contains data from individual countries. The accuracy and availability of the data may be affected by the quality of national registry data.

2. The study acknowledges the unknown number of undiagnosed cases of CKD in older adults, which may limit the analysis of the true burden of the disease.

3. There is a lack of detailed information on risk factors for CKD in older adults, which restricts the ability to analyze contributing factors comprehensively.

4. The GBD database categorizes CKD into five broad categories, including "chronic kidney disease due to other and unspecified causes." This classification may lead to wasted data and limit the study of specific etiologies and mechanisms of CKD.

Further, the study does not explicitly mention biases, but based on the limitations described, there some potential biases the authors should be aware of;

1. The study relies on data from the GBD database, which may not fully capture populations in countries with weaker data systems. This could lead to a selection bias and underrepresentation of certain regions or demographics.

2. Variability in the quality and completeness of national registry data may result in underreporting or misreporting of CKD cases.

3. The broad categorization of CKD causes (e.g., "other and unspecified causes") may obscure specific etiologies, leading to less precise analysis.

4. The study focuses on older adults aged 75 years and above, potentially overlooking trends and risk factors in younger age groups that could influence CKD progression in later years.

Addressing these biases in future research would require more granular data collection, improved classification systems, and efforts to include underrepresented populations.

**********

6. PLOS authors have the option to publish the peer review history of their article (what does this mean?). If published, this will include your full peer review and any attached files.

If you choose “no”, your identity will remain anonymous but your review may still be made public.

**Do you want your identity to be public for this peer review?**  For information about this choice, including consent withdrawal, please see our Privacy Policy.

Reviewer #1: No

Reviewer #2: **Yes:** U S H GAMAGE

**********

[NOTE: If reviewer comments were submitted as an attachment file, they will be attached to this email and accessible via the submission site. Please log into your account, locate the manuscript record, and check for the action link "View Attachments". If this link does not appear, there are no attachment files.]

While revising your submission, please upload your figure files to the Preflight Analysis and Conversion Engine (PACE) digital diagnostic tool, https://pacev2.apexcovantage.com/. PACE helps ensure that figures meet PLOS requirements. To use PACE, you must first register as a user. Registration is free. Then, login and navigate to the UPLOAD tab, where you will find detailed instructions on how to use the tool. If you encounter any issues or have any questions when using PACE, please email PLOS at figures@plos.org. Please note that Supporting Information files do not need this step.

# Author comment: Author response to Decision Letter 1

**DOI:** 10.1371/journal.pone.0354811.r002

18 Oct 2025

Thank you for your letter and for the reviewers’ comments concerning your manuscript entitled “Global, Regional, and National Burden of Chronic Kidney Disease in Older Adults From 1990 to 2021: Results From the Global Burden of Disease Study 2021”. Actually, as for the suggestions of the Reviewers, the revised manuscript I have submitted. Those comments are all valuable and very helpful for revising and improving our paper, as well as the important guiding significance to our researches. We have studied comments carefully and have made correction which we hope meet with approval.

Our submitted Response to Reviewers provides a point-by-point response to the comments raised by you and the reviewers. The relevant comments have been reproduced in full, with our replies in red text immediately following. We appreciate your permission to resubmit the revised manuscript. We hope the revised version meets the requirements and will be accepted for publication in PLOS ONE.

Attachment Submitted filename: Response to Reviewers.docx

# Peer review: Decision Letter 1

**DOI:** 10.1371/journal.pone.0354811.r003

**Authors:** Pasyodun Koralage Buddhika Mahesh

25 Dec 2025

PONE-D-25-28504R1

Global, Regional, and National Burden of Chronic Kidney Disease in Older Adults From 1990 to 2021: Results From the Global Burden of Disease Study 2021

PLOS One

Dear Dr. Shang,

Thank you for submitting your manuscript to PLOS ONE. After careful consideration, we feel that it has merit but does not fully meet PLOS ONE’s publication criteria as it currently stands. Therefore, we invite you to submit a revised version of the manuscript that addresses the points raised during the review process.

Please submit your revised manuscript by Feb 08 2026 11:59PM. If you will need more time than this to complete your revisions, please reply to this message or contact the journal office at plosone@plos.org. When you're ready to submit your revision, log on to https://www.editorialmanager.com/pone/ and select the 'Submissions Needing Revision' folder to locate your manuscript file.

Please include the following items when submitting your revised manuscript:

- A letter that responds to each point raised by the academic editor and reviewer(s). You should upload this letter as a separate file labeled 'Response to Reviewers'.
- A marked-up copy of your manuscript that highlights changes made to the original version. You should upload this as a separate file labeled 'Revised Manuscript with Track Changes'.
- An unmarked version of your revised paper without tracked changes. You should upload this as a separate file labeled 'Manuscript'.

-->If you would like to make changes to your financial disclosure, please include your updated statement in your cover letter. Guidelines for resubmitting your figure files are available below the reviewer comments at the end of this letter.

If applicable, we recommend that you deposit your laboratory protocols in protocols.io to enhance the reproducibility of your results. Protocols.io assigns your protocol its own identifier (DOI) so that it can be cited independently in the future. For instructions see: https://journals.plos.org/plosone/s/submission-guidelines#loc-laboratory-protocols. Additionally, PLOS ONE offers an option for publishing peer-reviewed Lab Protocol articles, which describe protocols hosted on protocols.io. Read more information on sharing protocols at https://plos.org/protocols?utm_medium=editorial-email&utm_source=authorletters&utm_campaign=protocols.

We look forward to receiving your revised manuscript.

Kind regards,

Pasyodun Koralage Buddhika Mahesh

Academic Editor

PLOS One

Journal Requirements:

1. If the reviewer comments include a recommendation to cite specific previously published works, please review and evaluate these publications to determine whether they are relevant and should be cited. There is no requirement to cite these works unless the editor has indicated otherwise.

2. Please review your reference list to ensure that it is complete and correct. If you have cited papers that have been retracted, please include the rationale for doing so in the manuscript text, or remove these references and replace them with relevant current references. Any changes to the reference list should be mentioned in the rebuttal letter that accompanies your revised manuscript. If you need to cite a retracted article, indicate the article’s retracted status in the References list and also include a citation and full reference for the retraction notice.

[Note: HTML markup is below. Please do not edit.]

Reviewers' comments:

Reviewer's Responses to Questions

**Comments to the Author**

1. If the authors have adequately addressed your comments raised in a previous round of review and you feel that this manuscript is now acceptable for publication, you may indicate that here to bypass the “Comments to the Author” section, enter your conflict of interest statement in the “Confidential to Editor” section, and submit your "Accept" recommendation.

Reviewer #2: All comments have been addressed

Reviewer #3: (No Response)

**********

2. Is the manuscript technically sound, and do the data support the conclusions?

The manuscript must describe a technically sound piece of scientific research with data that supports the conclusions. Experiments must have been conducted rigorously, with appropriate controls, replication, and sample sizes. The conclusions must be drawn appropriately based on the data presented.

Reviewer #2: Yes

Reviewer #3: Yes

**********

3. Has the statistical analysis been performed appropriately and rigorously?

Reviewer #2: Yes

Reviewer #3: Yes

**********

4. Have the authors made all data underlying the findings in their manuscript fully available?

The PLOS Data policy requires authors to make all data underlying the findings described in their manuscript fully available without restriction, with rare exception (please refer to the Data Availability Statement in the manuscript PDF file). The data should be provided as part of the manuscript or its supporting information, or deposited to a public repository. For example, in addition to summary statistics, the data points behind means, medians and variance measures should be available. If there are restrictions on publicly sharing data—e.g. participant privacy or use of data from a third party—those must be specified.

Reviewer #2: Yes

Reviewer #3: Yes

**********

5. Is the manuscript presented in an intelligible fashion and written in standard English?

PLOS ONE does not copyedit accepted manuscripts, so the language in submitted articles must be clear, correct, and unambiguous. Any typographical or grammatical errors should be corrected at revision, so please note any specific errors here.

Reviewer #2: Yes

Reviewer #3: Yes

**********

6. Review Comments to the Author

Please use the space provided to explain your answers to the questions above. You may also include additional comments for the author, including concerns about dual publication, research ethics, or publication ethics. (Please upload your review as an attachment if it exceeds 20,000 characters)

Reviewer #2: You have demonstrated a thorough and thoughtful response to the reviewers' comments, improving the manuscript's quality and addressing all concerns raised.

The authors made several specific changes in response to the reviewers' comments, as outlined in their response letter. Here are the key changes:

Formatting and Style Adjustments

1. Manuscript Formatting: Revised to meet PLOS ONE's style requirements, including file naming conventions and formatting guidelines.

2. Figure Captions: Separate captions were added for each figure to improve clarity.

3. Figure Legends: Updated to ensure readability, addressing concerns about the previous legends being unclear.

4. Number Formatting: Large numbers were formatted with thousand separators, and unnecessary decimals were removed for better visualization.

Clarifications in Results

1. Prevalence and Incidence Trends: The authors clarified the sentence in the results section of the abstract to avoid confusion. The revised text now accurately states:

"From 1990 to 2021, the prevalence of CKD in the older adults shows a decreasing trend, with EAPCs of -0.06 (95%UI, -0.09--0.03); the incidence of CKD in the older adults shows an increasing trend, with EAPCs of 0.50 (95%UI, 0.47-0.53)."

2. Redundant Phrases: Removed repeated phrases like "older adults" in the introduction section.

Expanded Discussion

1. Potential Causes of Findings: The discussion section was expanded to analyze the potential causes underlying the Estimated Annual Percentage Changes (EAPCs) findings, strengthening the analytical basis for conclusions.

2. Biases and Limitations: The limitations section was revised to explicitly address biases related to data accuracy, underreporting, and broad categorization in the GBD database. The authors acknowledged the challenges of relying on national registry data and the potential underrepresentation of certain demographics.

Response to Reviewer Suggestions

1. Clarification of Trends: The authors clarified trends in CKD prevalence and incidence in the abstract and results sections.

2. Improved Number Presentation: Removed decimals from large numbers and added thousand separators for better visualization.

3. Addressing Biases: Expanded the limitations section to include potential biases and challenges in data collection and analysis.

Acknowledgment of Reviewer Suggestions

1. Incorporation of Relevant References: The authors evaluated suggested references and incorporated them if deemed relevant.

2. Future Research Directions: Suggestions for addressing biases in future research were included, such as improved classification systems and efforts to include underrepresented populations.

These changes demonstrate that the authors carefully considered and addressed all reviewer comments, improving the manuscript's clarity, readability, and analytical depth.

Reviewer #3: The authors have made a clear effort to address the points raised in the previous round, and the manuscript is notably improved. The issues around formatting, clarity, and internal consistency have largely been resolved, and the paper is now much easier to read and interpret. In particular, the abstract has been corrected so that the trends in prevalence and incidence are no longer contradictory, the large numbers are formatted in a more reader-friendly way, repeated wording has been removed, and the figure legends are now legible.

I also appreciate the expanded Discussion and the more explicit limitations section. The authors now acknowledge the dependence on registry quality, the likelihood of underdiagnosis and underreporting, the lack of risk factor detail, the broad etiological categories used in the database, and the fact that focusing on people aged 75 and over may miss earlier determinants of disease. This transparency strengthens the paper and addresses most of the conceptual concerns raised previously.

That said, there are still a few points that I think would benefit from further clarification before publication.

1.The manuscript would benefit from a clearer statement of what is new here relative to existing global CKD burden studies. The focus on adults aged 75 years and older is important, but this is not highlighted strongly enough as the key contribution. I would encourage the authors to add one or two sentences in the Introduction or early Discussion explaining explicitly how this analysis differs from, and adds to, prior GBD-based work.

2.The description of the study population and age groups could be made more consistent. The Methods mention different concepts of “older” adults, but the analysis appears to focus on those aged 75 and above. This should be stated very clearly, and any background references to other age groups should be either clarified or removed to avoid confusion.

3.The statistical methods are described only briefly. While this is not a methods paper, readers would benefit from a little more detail on how EAPCs were estimated, whether crude or age-standardized rates were used for each outcome, and how uncertainty was handled. A short expansion of this section would improve reproducibility.

4.some of the interpretations of correlations (for example between SDI and DALYs or mortality) are plausible but somewhat strong given the ecological nature of the data. It would be helpful to add a short caution that these associations are not causal and may reflect confounding by healthcare access, diagnostic practices, survival, and reporting differences.

Related to this, there are places where levels and trends are discussed together in a way that could confuse readers for example, a region may have a high current burden but a slower growth rate, or vice versa. Making this distinction explicit in the text would improve clarity.

Finally, although the authors have explained the source and licensing of the map data in their response, it would be safer to include a brief attribution directly in the figure captions so that this information is visible in the published article.

A final light language edit would also help smooth some phrasing, but this is a minor point.

**********

7. PLOS authors have the option to publish the peer review history of their article (what does this mean?). If published, this will include your full peer review and any attached files.

If you choose “no”, your identity will remain anonymous but your review may still be made public.

**Do you want your identity to be public for this peer review?**  For information about this choice, including consent withdrawal, please see our Privacy Policy.

Reviewer #2: **Yes:** UDAYA SAMANTHILAKA HATTOTUWA GAMAGE

Reviewer #3: No

**********

[NOTE: If reviewer comments were submitted as an attachment file, they will be attached to this email and accessible via the submission site. Please log into your account, locate the manuscript record, and check for the action link "View Attachments". If this link does not appear, there are no attachment files.]

To ensure your figures meet our technical requirements, please review our figure guidelines: https://journals.plos.org/plosone/s/figures

You may also use PLOS’s free figure tool, NAAS, to help you prepare publication quality figures: https://journals.plos.org/plosone/s/figures#loc-tools-for-figure-preparation.

NAAS will assess whether your figures meet our technical requirements by comparing each figure against our figure specifications.

# Author comment: Author response to Decision Letter 2

**DOI:** 10.1371/journal.pone.0354811.r004

26 Jan 2026

Dear editors and reviewers, we are unable to present the detailed content of our revisions here, so we have included all our modification suggestions in the "Response to Reviewers" file.

Attachment Submitted filename: Response_to_Reviewers_auresp_2.docx

# Peer review: Decision Letter 2

**DOI:** 10.1371/journal.pone.0354811.r005

**Authors:** Pasyodun Koralage Buddhika Mahesh

25 Feb 2026

PONE-D-25-28504R2

Global, Regional, and National Burden of Chronic Kidney Disease in Older Adults From 1990 to 2021: Results From the Global Burden of Disease Study 2021

PLOS One

Dear Dr. Shang,

Thank you for submitting your manuscript to PLOS ONE. After careful consideration, we feel that it has merit but does not fully meet PLOS ONE’s publication criteria as it currently stands. Therefore, we invite you to submit a revised version of the manuscript that addresses the points raised during the review process.

Please submit your revised manuscript by Apr 11 2026 11:59PM. If you will need more time than this to complete your revisions, please reply to this message or contact the journal office at plosone@plos.org. When you're ready to submit your revision, log on to https://www.editorialmanager.com/pone/ and select the 'Submissions Needing Revision' folder to locate your manuscript file.

Please include the following items when submitting your revised manuscript:

- A letter that responds to each point raised by the academic editor and reviewer(s). You should upload this letter as a separate file labeled 'Response to Reviewers'.
- A marked-up copy of your manuscript that highlights changes made to the original version. You should upload this as a separate file labeled 'Revised Manuscript with Track Changes'.
- An unmarked version of your revised paper without tracked changes. You should upload this as a separate file labeled 'Manuscript'.

If you would like to make changes to your financial disclosure, please include your updated statement in your cover letter. Guidelines for resubmitting your figure files are available below the reviewer comments at the end of this letter.

If applicable, we recommend that you deposit your laboratory protocols in protocols.io to enhance the reproducibility of your results. Protocols.io assigns your protocol its own identifier (DOI) so that it can be cited independently in the future. For instructions see: https://journals.plos.org/plosone/s/submission-guidelines#loc-laboratory-protocols. Additionally, PLOS ONE offers an option for publishing peer-reviewed Lab Protocol articles, which describe protocols hosted on protocols.io. Read more information on sharing protocols at https://plos.org/protocols?utm_medium=editorial-email&utm_source=authorletters&utm_campaign=protocols.

We look forward to receiving your revised manuscript.

Kind regards,

Pasyodun Koralage Buddhika Mahesh

Academic Editor

PLOS One

Journal Requirements:

If the reviewer comments include a recommendation to cite specific previously published works, please review and evaluate these publications to determine whether they are relevant and should be cited. There is no requirement to cite these works unless the editor has indicated otherwise.

[Note: HTML markup is below. Please do not edit.]

Reviewers' comments:

Reviewer's Responses to Questions

**Comments to the Author**

1. If the authors have adequately addressed your comments raised in a previous round of review and you feel that this manuscript is now acceptable for publication, you may indicate that here to bypass the “Comments to the Author” section, enter your conflict of interest statement in the “Confidential to Editor” section, and submit your "Accept" recommendation.

Reviewer #3: (No Response)

**********

2. Is the manuscript technically sound, and do the data support the conclusions?

The manuscript must describe a technically sound piece of scientific research with data that supports the conclusions. Experiments must have been conducted rigorously, with appropriate controls, replication, and sample sizes. The conclusions must be drawn appropriately based on the data presented.

Reviewer #3: Partly

**********

3. Has the statistical analysis been performed appropriately and rigorously?

Reviewer #3: No

**********

4. Have the authors made all data underlying the findings in their manuscript fully available?

The PLOS Data policy requires authors to make all data underlying the findings described in their manuscript fully available without restriction, with rare exception (please refer to the Data Availability Statement in the manuscript PDF file). The data should be provided as part of the manuscript or its supporting information, or deposited to a public repository. For example, in addition to summary statistics, the data points behind means, medians and variance measures should be available. If there are restrictions on publicly sharing data—e.g. participant privacy or use of data from a third party—those must be specified.

Reviewer #3: Yes

**********

5. Is the manuscript presented in an intelligible fashion and written in standard English?

PLOS ONE does not copyedit accepted manuscripts, so the language in submitted articles must be clear, correct, and unambiguous. Any typographical or grammatical errors should be corrected at revision, so please note any specific errors here.

Reviewer #3: Yes

**********

6. Review Comments to the Author

Please use the space provided to explain your answers to the questions above. You may also include additional comments for the author, including concerns about dual publication, research ethics, or publication ethics. (Please upload your review as an attachment if it exceeds 20,000 characters)

Reviewer #3: Reviewer Report

Overall Evaluation

The authors have clearly made an effort to address the concerns raised in the previous round of review. The manuscript is improved in several respects. The definition of the study population is now clearer, the limitations section has been expanded appropriately, and the discussion of SDI-related findings is more cautious. Formatting issues have also been improved, and the additional explanation of the GBD data source and EAPC interpretation is helpful.

That said, despite these revisions, the manuscript still has substantive weaknesses that prevent it from being suitable for publication at this stage. In particular, there are ongoing concerns regarding methodological transparency, analytical depth, numerical consistency, and the overall strength of the scientific contribution. For these reasons, I recommend major revision.

Major Issues Requiring Attention

1. The study remains primarily descriptive and is essentially a subgroup analysis of publicly available GBD data limited to individuals aged 75 years and older. While this age-focused perspective is relevant, the manuscript does not convincingly demonstrate how this approach substantially advances existing knowledge from prior global CKD burden studies.

The analysis does not introduce new modeling strategies, decomposition approaches, or age–period–cohort analysis. Nor does it provide deeper mechanistic or policy-level insight beyond descriptive trends. The authors should strengthen the justification for the study’s added value and clarify what new understanding emerges specifically from focusing on this age group.

2. Although a section describing GBD methodology has been added, several important methodological points remain unclear:

Whether all reported rates are age-standardized, or if crude and standardized rates are mixed.

The exact regression specification used for EAPC estimation (e.g., log-linear modeling).

Whether assumptions underlying linear trend estimation were assessed.

How statistical uncertainty was incorporated into the analyses.

For transparency and reproducibility, these details should be explicitly described.

3. There are still numerical and typographical inconsistencies, including irregular formatting of large numbers and misplaced digits in some sections (including the abstract). These errors are concerning at the R2 stage and raise questions about data verification.

The authors should carefully recheck: Case numbers, Rates, Confidence intervals, EAPC values. A comprehensive numerical audit is necessary.

4. The Results section frequently enumerates country-level findings without sufficient analytical synthesis. This reduces the interpretive value of the manuscript and makes the section difficult to follow.

The authors should reduce repetitive listing of country statistics and instead emphasize broader patterns, regional contrasts, and key comparative insights. The current presentation reads more like a data summary than an analytical epidemiological study.

5. Although the discussion has been moderated, some passages still suggest explanatory mechanisms (particularly regarding SDI and aging) that go beyond what can be supported by ecological, descriptive data. Interpretations should remain clearly framed within the study design.

6. The manuscript would benefit from further language polishing. There are still grammatical inconsistencies and redundant phrasing that affect clarity.

**********

7. PLOS authors have the option to publish the peer review history of their article (what does this mean?). If published, this will include your full peer review and any attached files.

If you choose “no”, your identity will remain anonymous but your review may still be made public.

**Do you want your identity to be public for this peer review?**  For information about this choice, including consent withdrawal, please see our Privacy Policy.

Reviewer #3: No

**********

[NOTE: If reviewer comments were submitted as an attachment file, they will be attached to this email and accessible via the submission site. Please log into your account, locate the manuscript record, and check for the action link "View Attachments". If this link does not appear, there are no attachment files.]

To ensure your figures meet our technical requirements, please review our figure guidelines: https://journals.plos.org/plosone/s/figures

You may also use PLOS’s free figure tool, NAAS, to help you prepare publication quality figures: https://journals.plos.org/plosone/s/figures#loc-tools-for-figure-preparation.

NAAS will assess whether your figures meet our technical requirements by comparing each figure against our figure specifications.

# Author comment: Author response to Decision Letter 3

**DOI:** 10.1371/journal.pone.0354811.r006

14 Apr 2026

Dear Editor and Reviewers, we have made substantial revisions to the manuscript in order to meet the journal’s requirements and improve its readability. A detailed account of the revisions is provided in the file ‘Response to Reviewers’.

Attachment Submitted filename: Response_to_Reviewers_auresp_3.docx

# Peer review: Decision Letter 3

**DOI:** 10.1371/journal.pone.0354811.r007

**Authors:** Pasyodun Koralage Buddhika Mahesh

15 May 2026

PONE-D-25-28504R3

Global, Regional, and National Burden of Chronic Kidney Disease in Older Adults From 1990 to 2021: Results From the Global Burden of Disease Study 2021

PLOS One

Dear Dr. Shang,

Thank you for submitting your manuscript to PLOS ONE. After careful consideration, we feel that it has merit but does not fully meet PLOS ONE’s publication criteria as it currently stands. Therefore, we invite you to submit a revised version of the manuscript that addresses the points raised during the review process.

Please submit your revised manuscript by Jun 29 2026 11:59PM. If you will need more time than this to complete your revisions, please reply to this message or contact the journal office at plosone@plos.org. When you're ready to submit your revision, log on to https://www.editorialmanager.com/pone/ and select the 'Submissions Needing Revision' folder to locate your manuscript file.

Please include the following items when submitting your revised manuscript:

- A letter that responds to each point raised by the academic editor and reviewer(s). You should upload this letter as a separate file labeled 'Response to Reviewers'.
- A marked-up copy of your manuscript that highlights changes made to the original version. You should upload this as a separate file labeled 'Revised Manuscript with Track Changes'.
- An unmarked version of your revised paper without tracked changes. You should upload this as a separate file labeled 'Manuscript'.

If you would like to make changes to your financial disclosure, please include your updated statement in your cover letter. Guidelines for resubmitting your figure files are available below the reviewer comments at the end of this letter.

If applicable, we recommend that you deposit your laboratory protocols in protocols.io to enhance the reproducibility of your results. Protocols.io assigns your protocol its own identifier (DOI) so that it can be cited independently in the future. For instructions see: https://journals.plos.org/plosone/s/submission-guidelines#loc-laboratory-protocols. Additionally, PLOS ONE offers an option for publishing peer-reviewed Lab Protocol articles, which describe protocols hosted on protocols.io. Read more information on sharing protocols at https://plos.org/protocols?utm_medium=editorial-email&utm_source=authorletters&utm_campaign=protocols.

As the corresponding author, your ORCID iD is verified in the submission system and will appear in the published article. PLOS supports the use of ORCID, and we encourage all coauthors to register for an ORCID iD and use it as well. Please encourage your coauthors to verify their ORCID iD within the submission system before final acceptance, as unverified ORCID iDs will not appear in the published article. *Only*  the individual author can complete the verification step; PLOS staff *cannot*  verify ORCID iDs on behalf of authors.

We look forward to receiving your revised manuscript.

Kind regards,

Pasyodun Koralage Buddhika Mahesh

Academic Editor

PLOS One

Journal Requirements:

If the reviewer comments include a recommendation to cite specific previously published works, please review and evaluate these publications to determine whether they are relevant and should be cited. There is no requirement to cite these works unless the editor has indicated otherwise.

Please review your reference list to ensure that it is complete and correct. If you have cited papers that have been retracted, please include the rationale for doing so in the manuscript text, or remove these references and replace them with relevant current references. Any changes to the reference list should be mentioned in the rebuttal letter that accompanies your revised manuscript. If you need to cite a retracted article, indicate the article’s retracted status in the References list and also include a citation and full reference for the retraction notice.

[Note: HTML markup is below. Please do not edit.]

Reviewers' comments:

Reviewer's Responses to Questions

**Comments to the Author**

1. If the authors have adequately addressed your comments raised in a previous round of review and you feel that this manuscript is now acceptable for publication, you may indicate that here to bypass the “Comments to the Author” section, enter your conflict of interest statement in the “Confidential to Editor” section, and submit your "Accept" recommendation.

Reviewer #3: (No Response)

**********

2. Is the manuscript technically sound, and do the data support the conclusions?

The manuscript must describe a technically sound piece of scientific research with data that supports the conclusions. Experiments must have been conducted rigorously, with appropriate controls, replication, and sample sizes. The conclusions must be drawn appropriately based on the data presented.

Reviewer #3: Yes

**********

3. Has the statistical analysis been performed appropriately and rigorously?

Reviewer #3: Yes

**********

4. Have the authors made all data underlying the findings in their manuscript fully available?

The PLOS Data policy requires authors to make all data underlying the findings described in their manuscript fully available without restriction, with rare exception (please refer to the Data Availability Statement in the manuscript PDF file). The data should be provided as part of the manuscript or its supporting information, or deposited to a public repository. For example, in addition to summary statistics, the data points behind means, medians and variance measures should be available. If there are restrictions on publicly sharing data—e.g. participant privacy or use of data from a third party—those must be specified.

Reviewer #3: Yes

**********

5. Is the manuscript presented in an intelligible fashion and written in standard English?

PLOS ONE does not copyedit accepted manuscripts, so the language in submitted articles must be clear, correct, and unambiguous. Any typographical or grammatical errors should be corrected at revision, so please note any specific errors here.

Reviewer #3: Yes

**********

6. Review Comments to the Author

Please use the space provided to explain your answers to the questions above. You may also include additional comments for the author, including concerns about dual publication, research ethics, or publication ethics. (Please upload your review as an attachment if it exceeds 20,000 characters)

Reviewer #3: 1.The manuscript has improved significantly, especially in the methods and discussion sections, and most of the major reviewer concerns have been addressed well. The added explanations on data sources, statistical methods, and study limitations have strengthened the paper considerably.

2. The results section is clearer than before, but it still feels too descriptive in some parts, with many regional and country-specific examples. Focusing more on the key global patterns and reducing repetitive details would improve readability and overall impact.

3. The discussion is much stronger now, particularly in explaining important findings such as the prevalence–mortality divergence and SDI-related differences. However, some sections could still be shortened and simplified to make the main messages clearer.

4.While the language has improved, the manuscript would still benefit from careful final editing to correct awkward phrasing, reduce repetition, and improve sentence flow. This would make the paper easier to read.

**********

7. PLOS authors have the option to publish the peer review history of their article (what does this mean?). If published, this will include your full peer review and any attached files.

If you choose “no”, your identity will remain anonymous but your review may still be made public.

**Do you want your identity to be public for this peer review?**  For information about this choice, including consent withdrawal, please see our Privacy Policy.

Reviewer #3: No

**********

[NOTE: If reviewer comments were submitted as an attachment file, they will be attached to this email and accessible via the submission site. Please log into your account, locate the manuscript record, and check for the action link "View Attachments". If this link does not appear, there are no attachment files.]

To ensure your figures meet our technical requirements, please review our figure guidelines: https://journals.plos.org/plosone/s/figures

You may also use PLOS’s free figure tool, NAAS, to help you prepare publication quality figures: https://journals.plos.org/plosone/s/figures#loc-tools-for-figure-preparation.

NAAS will assess whether your figures meet our technical requirements by comparing each figure against our figure specifications.

# Author comment: Author response to Decision Letter 4

**DOI:** 10.1371/journal.pone.0354811.r008

1 Jun 2026

Thank you for your constructive feedback and the opportunity to revise our manuscript. We have addressed all comments and detailed our specific revisions in the Response to Reviewers below. Corresponding changes in the manuscript have been highlighted in red for your convenience.

Attachment Submitted filename: Response_to_Reviewers_auresp_4.docx

# Peer review: Decision Letter 4

**DOI:** 10.1371/journal.pone.0354811.r009

**Authors:** Pasyodun Koralage Buddhika Mahesh

14 Jul 2026

Global, Regional, and National Burden of Chronic Kidney Disease in Older Adults From 1990 to 2021: Results From the Global Burden of Disease Study 2021

PONE-D-25-28504R4

Dear Dr. Shang,

We’re pleased to inform you that your manuscript has been judged scientifically suitable for publication and will be formally accepted for publication once it meets all outstanding technical requirements.

Within one week, you’ll receive an e-mail detailing the required amendments. When these have been addressed, you’ll receive a formal acceptance letter and your manuscript will be scheduled for publication.

An invoice will be generated when your article is formally accepted. Please note, if your institution has a publishing partnership with PLOS and your article meets the relevant criteria, all or part of your publication costs will be covered. Please make sure your user information is up-to-date by logging into Editorial Manager at Editorial Manager® and clicking the ‘Update My Information' link at the top of the page. For questions related to billing, please contact billing support.

If your institution or institutions have a press office, please notify them about your upcoming paper to help maximize its impact. If they’ll be preparing press materials, please inform our press team as soon as possible -- no later than 48 hours after receiving the formal acceptance. Your manuscript will remain under strict press embargo until 2 pm Eastern Time on the date of publication. For more information, please contact onepress@plos.org.

Kind regards,

Pasyodun Koralage Buddhika Mahesh

Academic Editor

PLOS One

Additional Editor Comments (optional):

Reviewers' comments:

Reviewer's Responses to Questions

**Comments to the Author**

1. If the authors have adequately addressed your comments raised in a previous round of review and you feel that this manuscript is now acceptable for publication, you may indicate that here to bypass the “Comments to the Author” section, enter your conflict of interest statement in the “Confidential to Editor” section, and submit your "Accept" recommendation.

Reviewer #3: All comments have been addressed

**********

2. Is the manuscript technically sound, and do the data support the conclusions?

The manuscript must describe a technically sound piece of scientific research with data that supports the conclusions. Experiments must have been conducted rigorously, with appropriate controls, replication, and sample sizes. The conclusions must be drawn appropriately based on the data presented.

Reviewer #3: Yes

**********

3. Has the statistical analysis been performed appropriately and rigorously?

Reviewer #3: Yes

**********

4. Have the authors made all data underlying the findings in their manuscript fully available?

The PLOS Data policy requires authors to make all data underlying the findings described in their manuscript fully available without restriction, with rare exception (please refer to the Data Availability Statement in the manuscript PDF file). The data should be provided as part of the manuscript or its supporting information, or deposited to a public repository. For example, in addition to summary statistics, the data points behind means, medians and variance measures should be available. If there are restrictions on publicly sharing data—e.g. participant privacy or use of data from a third party—those must be specified.

Reviewer #3: Yes

**********

5. Is the manuscript presented in an intelligible fashion and written in standard English?

PLOS ONE does not copyedit accepted manuscripts, so the language in submitted articles must be clear, correct, and unambiguous. Any typographical or grammatical errors should be corrected at revision, so please note any specific errors here.

Reviewer #3: Yes

**********

6. Review Comments to the Author

Please use the space provided to explain your answers to the questions above. You may also include additional comments for the author, including concerns about dual publication, research ethics, or publication ethics. (Please upload your review as an attachment if it exceeds 20,000 characters)

Reviewer #3: This manuscript offers a detailed global assessment of chronic kidney disease (CKD) among adults aged 75 years and older, using data from the GBD 2021 study. The authors have made strong improvements in the revised version. The methods are clearer, the Results section is more focused, and the Discussion now highlights the main findings more directly. Previous reviewer concerns have been addressed well, and the manuscript reads more smoothly overall.

The study is relevant, well‑designed, and adds important evidence about CKD burden in the oldest age groups.

Minor Revision

The manuscript uses “older adults,” “elderly,” and “oldest‑old” at different points. Standardising terminology; preferably “older adults”; would improve clarity.

**********

7. PLOS authors have the option to publish the peer review history of their article (what does this mean?). If published, this will include your full peer review and any attached files.

If you choose “no”, your identity will remain anonymous but your review may still be made public.

**Do you want your identity to be public for this peer review?**  For information about this choice, including consent withdrawal, please see our Privacy Policy.

Reviewer #3: No

**********

# Editor report: Acceptance letter

**DOI:** 10.1371/journal.pone.0354811.r010

**Authors:** Pasyodun Koralage Buddhika Mahesh

PONE-D-25-28504R4

PLOS One

Dear Dr. Shang,

I'm pleased to inform you that your manuscript has been deemed suitable for publication in PLOS One. Congratulations! Your manuscript is now being handed over to our production team.

At this stage, our production department will prepare your paper for publication. This includes ensuring the following:

* All references, tables, and figures are properly cited

* All relevant supporting information is included in the manuscript submission,

* There are no issues that prevent the paper from being properly typeset

You will receive further instructions from the production team, including instructions on how to review your proof when it is ready. Please keep in mind that we are working through a large volume of accepted articles, so please give us a few days to review your paper and let you know the next and final steps.

Lastly, if your institution or institutions have a press office, please let them know about your upcoming paper now to help maximize its impact. If they'll be preparing press materials, please inform our press team within the next 48 hours. Your manuscript will remain under strict press embargo until 2 pm Eastern Time on the date of publication. For more information, please contact onepress@plos.org.

You will receive an invoice from PLOS for your publication fee after your manuscript has reached the completed accept phase. If you receive an email requesting payment before acceptance or for any other service, this may be a phishing scheme. Learn how to identify phishing emails and protect your accounts at https://explore.plos.org/phishing.

If we can help with anything else, please email us at customercare@plos.org.

Thank you for submitting your work to PLOS ONE and supporting open access.

Kind regards,

PLOS ONE Editorial Office Staff

on behalf of

Dr. Pasyodun Koralage Buddhika Mahesh

Academic Editor

PLOS One
